# Epilepsy in a mouse model of GNB1 encephalopathy arises from altered potassium (GIRK) channel signaling and is alleviated by a GIRK inhibitor

**DOI:** 10.3389/fncel.2023.1175895

**Published:** 2023-05-18

**Authors:** Sophie Colombo, Haritha P. Reddy, Sabrina Petri, Damian J. Williams, Boris Shalomov, Ryan S. Dhindsa, Sahar Gelfman, Daniel Krizay, Amal K. Bera, Mu Yang, Yueqing Peng, Christopher D. Makinson, Michael J. Boland, Wayne N. Frankel, David B. Goldstein, Nathan Dascal

**Affiliations:** ^1^Institute for Genomic Medicine, Columbia University Irving Medical Center, New York, NY, United States; ^2^Department of Physiology and Pharmacology, School of Medicine, Tel Aviv University, Tel Aviv, Israel; ^3^Department of Biotechnology, Bhupat and Jyoti Mehta School of Biosciences, Indian Institute of Technology Madras, Chennai, India; ^4^Mouse NeuroBehavior Core Facility, Columbia University Irving Medical Center, New York, NY, United States; ^5^Department of Pathology and Cell Biology, Columbia University Irving Medical Center, New York, NY, United States; ^6^Department of Neurology, Columbia University Irving Medical Center, New York, NY, United States; ^7^Department of Neuroscience, Columbia University, New York, NY, United States; ^8^Department of Genetics and Development, Columbia University Irving Medical Center, New York, NY, United States

**Keywords:** GNB1, G protein, animal model, neurodevelopmental disorder, spike-wave discharges, GIRK, absence seizures, ethosuximide

## Abstract

*De novo* mutations in *GNB1*, encoding the G*β*_1_ subunit of G proteins, cause a neurodevelopmental disorder with global developmental delay and epilepsy, *GNB1* encephalopathy. Here, we show that mice carrying a pathogenic mutation, K78R, recapitulate aspects of the disorder, including developmental delay and generalized seizures. Cultured mutant cortical neurons also display aberrant bursting activity on multi-electrode arrays. Strikingly, the antiepileptic drug ethosuximide (ETX) restores normal neuronal network behavior *in vitro* and suppresses spike-and-wave discharges (SWD) *in vivo*. ETX is a known blocker of T-type voltage-gated Ca^2+^ channels and G protein-coupled potassium (GIRK) channels. Accordingly, we present evidence that K78R results in a gain-of-function (GoF) effect by increasing the activation of GIRK channels in cultured neurons and a heterologous model (*Xenopus* oocytes)—an effect we show can be potently inhibited by ETX. This work implicates a GoF mechanism for GIRK channels in epilepsy, identifies a new mechanism of action for ETX in preventing seizures, and establishes this mouse model as a pre-clinical tool for translational research with predicative value for *GNB1* encephalopathy.

## Introduction

G protein-coupled receptors (GPCRs) constitute the largest family of transmembrane receptors, regulating many aspects of human physiology (Rosenbaum et al., 2009). Most GPCR signaling is transduced through a G protein heterotrimeric complex, composed of three subunits, Gα, G*β*, and Gγ. GPCR activation catalyzes a GDP-to-GTP exchange on the Gα subunit, leading to the dissociation of Gα from the obligate dimer G*βγ* (Oldham and Hamm, 2008; Smrcka, 2008). Both Gα and G*βγ* then bind and regulate a wide variety of downstream effectors (Smrcka, 2008; Khan et al., 2013). The G*β* subunits are encoded by five genes (*GNB1* to *GNB5*) in the human genome (Downes and Gautam, 1999). The G*β*_1−4_ subunits share 80–90% sequence similarity (Gautam et al., 1998), are differentially expressed across tissues, and trigger a variety of specialized cellular responses (Tennakoon et al., 2021). The less-homologous G*β*_5_ gene is expressed mostly in the nervous system (Howlett et al., 2009). Mutations affecting the activity of G*β* are linked to abnormal physiological functioning, resulting in disease (Malerba et al., 2019). Prior investigation of homozygous *Gnb1* mutant mice demonstrated that *Gnb1* is required for proper neurogenesis as *Gnb1* knockout embryos developed neural tube defects or exhibited microcephaly and neonatal lethality (Okae and Iwakura, 2010). Furthermore, *Gnb1* heterozygous mice had aberrant retina morphology and progressive degeneration.

Heterozygous *de novo GNB1* frameshift and pathogenic splice-site and missense mutations in humans cause MRD42 (MIM 139380), an autosomal dominant neurodevelopmental disorder (also known as *GNB1* encephalopathy) generally characterized by intellectual impairment, global developmental delay, hypotonia typically coupled with limb hypertonia, poor overall growth, and various manifestations of epilepsy in nearly 60% of patients (Petrovski et al., 2016; Lohmann et al., 2017; Hemati et al., 2018; Endo et al., 2020; Revah-Politi et al., 2020). Seizures usually appear at 1–4 years postnatally and can include tonic, absence, myoclonic, generalized tonic-clonic, and focal seizures, as well as epileptic spasms (Petrovski et al., 2016; Revah-Politi et al., 2020), which often result in detrimental and potentially irreversible effects (Berg, 2011). In some cases, attention-deficit hyperactivity disorder, nystagmus, strabismus, cortical visual impairment, and autistic characteristics were observed (Petrovski et al., 2016; Revah-Politi et al., 2020). *GNB1* mutations have been found in several tens of cases so far (Lansdon and Saunders, 2021; RoŽmarić et al., 2022), and approximately 75% of *GNB1* mutations affect residues in exon 6 or exon 7.

G*βγ* directly regulates neuronal excitability *via* several pathways: inhibition of voltage-gated calcium channels (Ikeda, 1996; Dolphin, 1998; Zamponi and Currie, 2013), activation of postsynaptic G protein-coupled inwardly rectifying potassium (GIRK) channels (Dascal, 1997; Lüscher and Slesinger, 2010; Malik and Johnston, 2017; Luo et al., 2022), and inhibition of vesicle release through interactions with the SNARE complex (Yoon et al., 2007; Zurawski et al., 2017). The breadth and complexity of G*βγ*-mediated signaling pathways thus represent a significant challenge to the understanding of GNB1-related disease mechanisms. As such, the mechanisms of action of *GNB1* mutations are generally not well understood. So far, few functional studies have been performed. In one study, authors investigated 10 *GNB1* mutations and observed altered G*βγ* dimer or Gα*βγ* heterotrimer formation and BRET induction in seven of them (Lohmann et al., 2017). Another study showed that some somatic *GNB1* mutations linked to cancer have a gain-of-function (GoF) effect in certain downstream signaling pathways (Yoda et al., 2015). In a recent study, inspired by the results of the present article published on a preprint server (Colombo et al., 2019), we investigated the effects of three *GNB1* mutations seen in patients with *GNB1* encephalopathy—K78R, I80N, and I80T—in heterologous expression systems. We demonstrated that these mutations did not alter GPCR-G_i/o_ protein coupling but had a significant effect on G*βγ* regulation of GIRK channels (Reddy et al., 2021).

To elucidate how *GNB1* mutations cause neurodevelopmental disease in a physiological context, we generated a mouse model of the pathogenic missense variant, K78R. We show that heterozygous mice (*Gnb*1^K78R/+^) recapitulate many clinical features of affected individuals, including developmental delay, motor and cognitive deficits, and absence-like generalized seizures. Cortical neurons from *Gnb*1^K78R/+^ mice cultured *in vitro* display several bursting phenotypes and an increase in GABA_B_ receptor-induced GIRK activity. We found that both the *in vivo* and *in vitro* excitability phenotypes are effectively suppressed by the anti-epileptic drug, ethosuximide (ETX). Finally, we expressed wild-type and mutant G*β*_1_ with GIRK1/2 and GIRK2 channels in Xenopus oocytes and established that the K78R mutation increases GIRK activation and the ETX exerts its effects, in part, through GIRK channel inhibition.

## Materials and methods

### Animals

#### Mice

*Gnb*1^*K*78*R*/+^ mice were generated using CRISPR/Cas9 at the Transgenics Mouse Shared Resources at Columbia University Irving Medical Center on a C57BL/6NCrl background. Mice were further backcrossed to C57BL/6NJ mice (B6NJ; JAX stock # 005304). For certain experiments, we used F1 Hybrid (F1H) mice obtained from a C57BL/6NJ x FVB.129P2 (JAX stock # 004828) mating (as indicated in the text). Wild-type (WT) littermates were used as controls. Mice were maintained in ventilated cages at controlled temperature (22–23°C), humidity ~60%, and 12h:12h light:dark cycles. Mice had access to regular chow and water, *ad libitum*. Mice were bred and procedures were conducted at the Columbia University Institute of Comparative Medicine, which is fully accredited by the Association for Assessment and Accreditation of Laboratory Animal Care, and were approved by the Columbia Institutional Animal Care and Use Committee (protocols #AC-AAAU8484, #AC-AAAZ8450, #AC-AAAR4414, and #AC-AAAU8476).

#### Xenopus laevis

Experiments were performed in accordance with relevant guidelines and regulations and approved by Tel Aviv University Institutional Animal Care and Use Committee (permits 01-16-104 and M-13-002). *Xenopus laevis* female frogs were maintained and operated as described (Dascal and Lotan, 1992; Hedin et al., 1996). Frogs were kept in dechlorinated water tanks at 20 ± 2°C on a 10-h light/14-h dark cycle, anesthetized in a 0.17–0.25% solution of procaine methanesulphonate (MS222), and portions of ovary were removed through a small incision in the abdomen. The incision was sutured, and the animal was held in a separate tank until it had fully recovered from the anesthesia and then returned to the post-operational animals' tank. The animals did not show any signs of post-operational distress and were allowed to recover for at least 3 months until the next surgery. Following the final collection of oocytes, after four surgeries at most, anesthetized frogs were killed by decapitation and double pithing.

### Genotyping of mice

DNA was extracted from the tail or ear tissue, and PCR was performed using the KAPA Mouse Genotyping Standard Kit (KAPA Biosystems). The following primers were used for PCR. Fwd: CGAGCATTGAGATCCTCTTTCT; Rev: GTCATCATTGCTCCATCAACAG. The restriction enzyme *HinfI* was used to distinguish WT from *Gnb*1^K78R/+^ mice.

### Protein extraction, Western blots, and immunocytochemistry in mice

These procedures are described in Supplementary material.

### Behavioral experiments

All behavioral experiments were performed in the Mouse Neurobehavioral Core facility at Columbia University Irving Medical Center.

#### Neonatal pup development

Pups are tattooed on the paws for identification on P2. Developmental milestones and pup vocalizations are conducted on alternated days to avoid over-handling.

##### Developmental milestones

We measured body weight, righting reflex (the latency to right oneself from a belly-up position to be on all four), negative geotaxis (on a wire mesh screen, the latency to turn 90° or 180° from a downward-facing start position), and vertical screen holding (the latency to fall off a vertically positioned wire mesh screen). To begin the test, each pup was gently removed from the nest and placed on a clean piece of bench protector. The cage lid was immediately and gently placed back, to reduce agitation in the nest. All assessment was completed by a deft experimenter within 3 min. At the end of the session, the pup quickly returned to the nest.

##### Ultrasonic vocalizations

Neonatal mouse pups emit ultrasonic vocalizations (USV) when separated from the dam. Separation-induced vocalizations were tested on postnatal days P5, P7, P9, and P11. The pup was gently removed from the nest and placed in a plastic container (10 cm × 8 cm × 8.5 cm) the bottom of which was covered with a 0.5-cm layer of fresh bedding. The cage lid was immediately and gently placed back, to avoid agitating the dam and the pups in the nest. The container holding the isolated pup was immediately placed inside a sound-attenuating environmental chamber (Med Associates). At the end of the 3 min recording session, each pup was marked (to avoid repeated handling on the same day) and returned to the nest. The procedure was repeated with all the pups of the same litter. Ultrasonic vocalizations were recorded by an Ultrasound Microphone (Avisoft UltraSoundGate condenser microphone CM16, Avisoft Bioacoustics) sensitive to frequencies of 10–180 kHz. Ultrasonic calls were recorded using the Avisoft Recorder software and analyzed using the Avisoft SASLab Pro software.

#### Adult motor tests

Motor tests were done on 3–4 month-old mice.

##### Open field exploratory activity

The open field test is the most commonly used general test for locomotor activity. Each mouse was gently placed in the center of a clear Plexiglas arena (27 × 27 × 20 cm, Med Associates ENV-510) lit with dim light (~5 lux), and allowed to ambulate freely for 60 min. Infrared (IR) beams embedded along the X, Y, and Z axes of the arena automatically tracked distance moved, horizontal movement, vertical movement, stereotypies, and time spent in the center zone. At the end of the test, the mouse was returned to the home cage, and the arena was cleaned with 70% ethanol followed by water, and wiped dry.

##### Rotarod

Motor learning was assessed using a mouse-accelerating RotaRod (Ugo Basile). Mice were placed on the rotating drum that accelerated from 5 to 40 rpm over 5 min. Mice were tested for three trials a day, for 2 consecutive days. The inter-trial interval was 1 h. Rotarod scores were used for latency to fall or ride the rod around for all three cohorts.

#### Adult learning and memory tests

##### Water maze acquisition and reversal

Spatial learning and reversal learning were assessed in the Morris water maze on 5–6 month-old mice using procedures and equipment as previously described (Yang et al., 2012) and are detailed in Supplementary methods.

##### Fear conditioning

This is a classic test for conditioned learning. Training and conditioning tests are conducted in two identical chambers (Med Associates) that were calibrated to deliver identical foot shocks. Each chamber was 30 cm × 24 cm × 21 cm with a clear polycarbonate front wall, two stainless side walls, and a white opaque back wall. The bottom of the chamber consisted of a removable grid floor with a waste pan underneath. When placed in the chamber, the grid floor is connected to a circuit board for delivery of a scrambled electric shock. Each conditioning chamber was placed inside a sound-attenuating environmental chamber (Med Associates). A camera mounted on the front door of the environmental chamber recorded test sessions which were later scored automatically, using the VideoFreeze software (Med Associates). For the training session, each chamber was illuminated with a white house light. An olfactory cue was added by dabbing a drop of imitation almond flavoring solution (1:100 dilution in water) on the metal tray beneath the grid floor. The mouse was placed in the test chamber and allowed to explore freely for 2 min. A pure tone (5 kHz, 80 dB) which served as the conditioned stimulus (CS) was played for 30 s. During the last 2 s of the tone, a foot shock (0.5 mA) was delivered as the unconditioned stimulus (US). Each mouse received three CS-US pairings, separated by 90 s intervals. After the last CS-US pairing, the mouse was left in the chamber for another 120 s, during which freezing behavior was observed by the VideoFreeze software. The mouse was then returned to its home cage. Contextual conditioning was tested 24 h later in the same chamber, with the same illumination and olfactory cue present but without foot shock. Each mouse was placed in the chamber for 5 min, in the absence of CS and US, during which freezing was measured. The mouse was then returned to its home cage. Cued conditioning was conducted 48 h after training. Contextual cues were altered by covering the grid floor with a smooth white plastic sheet, inserting a piece of black plastic sheet bent to form a vaulted ceiling, using near-infrared light instead of white light, and dabbing vanilla instead of almond odor on the floor. The session consisted of a 3-min free exploration period followed by 3 min of the identical CS tone (5 kHz, 80 dB). Freezing was scored during both 3 min segments. The mouse was then returned to its home cage. The chamber was thoroughly cleaned of odors between sessions, using 70% ethanol and water.

### Electroconvulsive threshold (ECT)

ECT tests were performed as previously published with modifications (Frankel et al., 2001; Kapur et al., 2020). In brief, tests were performed on experimentally naive mice beginning at 6–8 weeks of age. A drop of anesthetic containing 0.5% tetracaine and 0.9% NaCl was placed onto each eye and a fixed electrical current was applied via silver transcorneal electrodes using a customized electroconvulsive stimulator (Ugo Basile model 57800-D01, Stoelting Co., Wood Dale, IL). The stimulator was set to produce rectangular wave pulses with the following parameters: 299 Hz, 0.2 s duration, and 1.6 ms pulse width.

To find the generalized tonic-clonic seizure threshold (also previously described as “minimal clonic forebrain seizure”), the seizure response was determined for a particular current, beginning with a current approximately 0.5–1.0 mA below the known average threshold for that genetic background and sex. Seizures were scored using a modified Racine scale (seizure scores: 0, no observable symptoms; 1, stun; 2, myoclonic jerk/twitch; 3, rearing, forelimb and jaw clonus; 4, forelimb tonic extension; 5, tonic hindlimb extension). The current was increased in 0.5 to 1.0 mA increments daily until the mouse had a first clear seizure response (a score of 3 or higher). As male and female mice have different thresholds, the sexes were analyzed separately. For analysis, the mean integrated root mean square (iRMS) current is reported for each genotype-sex group. Group means were calculated to determine the threshold.

### Electroencephalogram (EEG)

For initial EEG screening, electrode implantation of adult mice approximately 6–8 weeks old and video-EEG was performed essentially as described (Asinof et al., 2016). Mice were anesthetized with tribromoethanol (250 mg/kg i.p., Sigma-Aldrich #T48402). Three small burr holes were drilled in the skull (1 mm rostral to the bregma on both sides and 2 mm caudal to the bregma on the left), 2 mm lateral to the midline. One hole was drilled over the cerebellum as a reference. Using four Teflon-coated silver wires soldered onto the pins of a microconnector (Mouser electronics #575- 501101), the wires were placed between the dura and the brain, and a dental cap was then applied. The mice were given a post-operative analgesic of carprofen (5 mg/kg subcutaneous Rimadyl injectable) and allowed a 48-h recovery period before recordings were taken. To record EEG signals, mice were connected with flexible recording cables to allow minimally restrictive movements within the cage. Signal (200 samples/s) was acquired either on a Grael II EEG amplifier (Compumedics) or on a Natus Quantum amplifier (Natus Neuro), and the data were analyzed either with the Profusion 5 (Compumedics) or NeuroWorks (Natus Neuro) software. Differential amplification recordings were recorded pair-wise between all three electrodes, as well as referential, providing a montage of six channels for each mouse. Mouse activity was captured simultaneously by video monitoring using a Sony IPELA EP550 model camera, with an infrared light to allow recordings in the dark. For initial screening and SWD event scoring, we recorded continuously for 24 to 48 h. For drug dosing studies, mice were recorded continuously for 48 h, injected with saline around 12 pm on day 1 and injected with the drug around 12 pm on day 2.

To analyze the effect of SWD events on movement, a custom-built MATLAB software was developed to perform real-time video-tracking while simultaneously conducting EEG recording on a Neuralynx Digital Lynx system. The software synced video-taping with EEG recording through a NetCom API provided by Neuralynx. An infrared camera was used to track the body position (the center of the whole body) of a mouse by subtracting each video frame from the background image, captured in the absence of the mouse. The animal's movement was calculated as the pixel distance between body positions divided by the time. Then, movement during SWD periods (detected by the algorithm described above) was averaged for each SWD duration or each inter-SWD interval, and further normalized for each animal to the average movement over the whole recording session.

### Multi-electrode array (MEA)

#### Plate preparation

One to 7 days before dissection, 48-well MEA plates (Axion Biosystems #M768-KAP-48) were coated with 50 μg/mL poly-D-lysine (Sigma-Aldrich #P0899-50MG) in borate buffer, then washed three times with phosphate-buffered saline (PBS) and stored in PBS at 4°C until use. Before use, PBS was aspirated and plates were allowed to dry in a sterilized hood.

#### Primary neuron culture

Cortical neurons were dissociated from the brains of postnatal day 0 (P0) C57BL/6NJ WT or *Gnb*1^K78R/+^ mice. Pups were immediately decapitated, weighed, and genotyped. The entire cerebral cortex was rapidly dissected and cut into small pieces under sterile conditions in cold Hibernate A solution (Gibco, #A1247501). Cortices from two WT or *Gnb*1^K78R/+^ pups were pooled together. The dissected cortices were then enzymatically digested in 20 U/mL Papain plus DNAse (Worthington Biochemical Corporation, #LK003178 and #LK003172) diluted in Hibernate A for 20 min at 37°C. Cells were centrifuged at 300 x*g* for 5 min, then the digestion was neutralized by aspirating the supernatant and adding a warm Hibernate A medium. Cells were mechanically dissociated by trituration and counted using a hemocytometer with a Trypan Blue counterstain. Cells were centrifuged at 300 x*g* for 5 min and resuspended at a density of 6,000 cells/μL in warm Neurobasal-A (Gibco #10888022), 1x B27 supplement (Gibco #17504044), 1x GlutaMax (Gibco #35050061), 1x MEM-NEAA (Gibco #11140050), 1% HEPES (Gibco #15630080), 1% penicillin/streptomycin (Gibco #15140122), 1% fetal bovine serum (Gibco #26140079), and 5 μg/mL laminin (Sigma-Aldrich #L2020). A total of 150,000 cells were plated on a pre-coated 48-well MEA plate in a 25-μL drop. The day after plating (DIV1), 100% of the media was removed and replaced with warm Neurobasal-A, 1x B27 supplement, 1x GlutaMax, 1% HEPES, and 1% penicillin/streptomycin (NBA/B27 medium). Glial growth was not chemically suppressed. Cultures were maintained at 37°C in 5% CO_2_. The medium was 50% changed every other day with fresh warm NBA/B27 starting on DIV3, after each recording session.

#### Data analysis of spontaneous recordings

MEA recordings were conducted on media change days before media change starting on DIV5. Plates were equilibrated for 5 min and then recorded for 15 min per day using Axion Biosystems Maestro 768 channel amplifier at 37°C in a CO_2_ gas-controlled chamber and Axion Integrated Studios (AxIS) software v2.4. Each well on a 48-well plate is comprised of 16 electrodes on a 4 by 4 grid with each electrode capturing the activity of nearby neurons. A Butterworth band-pass filter (200–3000 Hz) and an adaptive threshold spike detector set at 7x the standard deviation of the noise were used during recordings. Raw data and spike list files were collected. Spike list files were used to extract additional spike, burst, and network features using a custom MEA analysis software package for interpretation of neuronal activity patterns, meaRtools, based on rigorous permutation statistics that enable enhanced identification of over 70 activity features (Gelfman et al., 2018). Specifically, we analyzed spiking and bursting rates, spike density in bursts, periodicity of bursting, burst duration, and the time between bursts (i.e., inter-burst interval, IBI), as well as the synchronicity of the network. We determined the parameters for detecting neuronal bursts and network events based on published reports and experimentation (McConnell et al., 2012; Mack et al., 2014). Activity data were inspected to remove inactive electrodes and wells. For an electrode to be considered active, we required that at least five spikes per minute were recorded. Wells in which fewer than four electrodes were active for >50% of the days of recording were considered inactive and removed from analyses. For synchronous network events, at least five electrodes (>25% of the total in a well) were required to participate in a network event for it to qualify as a network spike or burst. Events with fewer participating electrodes were filtered. Bursts were detected using the Maximum Interval burst detection algorithm (Neuroexplorer software, Nex Technologies) implemented in the meaRtools package. We required that a burst consists of at least five spikes and lasts at least 0.05 s, and that the maximum duration between two spikes within a burst be 0.1 s at the beginning of a burst and 0.25 s at the end of a burst. Adjacent bursts were further merged if the duration between them is <0.8 s. These parameters were chosen based on the literature and in-house experimentation (Mack et al., 2014). To analyze data over time, we performed permuted Mann–Whitney U-tests. The values for each well for the chosen DIVs were combined and a Mann–Whitney U (MWU)-test was performed. The labels for each well (WT vs. *Gnb*1^K78R/+^) were then shuffled and permuted 1,000 times to create 1,000 datasets that were tested for significance using the MWU test. We report the permuted *p*-values as the rank of the true *p*-value within the distribution of permuted *p*-values. As mentioned, the *Gnb*1^K78R/+^ well data of each plate was normalized to the WT wells, before combining the plates for statistical analysis using an R script developed in-house. To normalize the data for each plate, we computed the average value among WT wells per DIV and then divided the values of each well per DIV by this resulting mean. For comparison, we also combined the individual *p*-values of each plate using an R script that performs the Fisher's method.

#### Pharmacology

Pharmacological studies were performed on mature cultures between DIV20 and DIV45. Media volumes were equilibrated to 300 μL per well. MEA plates were dosed by adding a defined amount of a stock concentration of drug to a parallel dosing plate, then 150 μL of the media from the MEA plate was removed and added to the parallel dosing plate and mixed before adding the media back to the MEA plate. Spontaneous activity in physiological solution was recorded for 3 min after equilibrating the MEA plates on the Maestro system for 10 min (baseline condition). Then, networks were exposed to the drug and spontaneous activity was recorded 10 min after drug application, for 3 min (drug condition). Results are presented as a ratio drug condition/baseline condition (baseline condition = 1, blue dashed line on graphs in Figures 3–5). To compare the effects of the drugs between WT and *Gnb*1^K78R/+^ neurons, we performed an MWU test with Bonferroni correction. For the chronic dosing of ETX from DIV3 to DIV31, ETX was added during medium change every other day. It was diluted in pre-warmed NBA/B27 just before the medium change to a final concentration of 250 μM, 750 μM, or 2 mM from a 200-mM stock solution.

### Drugs

All drugs used were purchased from commercial sources: ethosuximide (Sigma-Aldrich), valproate sodium (Sigma-Aldrich), phenytoin (Selleckchem), baclofen (Tocris), and bicuculline (Tocris or Hello Bio). For mouse injections, ethosuximide and valproate sodium were resuspended in 0.9% saline and phenytoin in 2% N,N-dimethylacetamide/10% propylene glycol/30% 2-hydroxypropyl-beta-cyclodextrin in H_2_O, and administered via intra-peritoneal (IP) injections in a volume of 10 ml/kg. For MEA, drugs were either resuspended in H_2_O for ethosuximide, valproate sodium, and baclofen or in DMSO for phenytoin. DMSO concentration on MEA never exceeded 0.1%. Concentrations used are reported in figure legends.

### Electrophysiology in primary neurons

Primary cortical and hippocampal neurons were maintained on a monolayer of mitotically inactivated primary astrocytes. To generate primary astrocytes, we modified a previously described protocol (Schildge et al., 2013) as follows. Cortices from P0 WT mice were dissociated as described above. Cell suspensions were plated onto 75 cm^2^ flasks pre-coated with poly-D-lysine (15 μg/mL, Sigma P6407) in astrocyte maintenance medium consisting of DMEM/F12 (Gibco), 10% heat-inactivated FBS (Gibco), 1x MEM-NEAA (Gibco), 1x glutamine (Gibco), and 1% penicillin/streptomycin (Gibco). Cultures were maintained in a humidified environment at 37°C in 5% CO_2_. The medium was changed every other day. Once confluent, flasks were tapped vigorously for 1 min to detach microglia, oligodendrocytes, and neurons, and the medium was aspirated. The remaining adherent cells were washed with 1x PBS and passaged with 0.05% trypsin (Gibco) and re-plated in fresh flasks pre-coated with PDL. This process was repeated three times to generate a homogenous culture of astrocytes. The absence of neurons was confirmed by staining for MAP2 (Sigma #M4403). On the final passage, astrocytes were mitotically inactivated by treatment with 10 μM cytosine *β*-D-arabinofuranoside (Ara-C, Sigma #C6645) for 6 h, washed two times with 1x PBS, and cryopreserved in cold maintenance medium containing 10% DMSO (Gibco) at 500,000 cells per vial.

Mitotically inactive astrocytes were seeded in an astrocyte maintenance medium onto 15 mm coverslips (50,000 cells per coverslip) pre-coated with 50 μg/mL PDL. The medium was changed every other day. After 7 days (DIV7), cortical and hippocampal neurons were prepared from P0 WT and *Gnb*1^K78R/+^ pups as described above. Neuronal preparations were plated at 75,000 cells per 15 mm coverslip containing primary astrocytes. Cultures were then maintained in Neurobasal-A, 1x B27 supplement, 1x GlutaMax, 1x MEM-NEAA, 1% HEPES, and 1% penicillin/streptomycin (NBA/B27 medium), and 50% medium was changed every other day with fresh NBA/B27 medium. On DIV8, cultures were transduced with 50,000 vg/cell of either AAV8-CaMKIIa-GFP (UNC Vector Core) or AAV9-mDlx-NLS-mRuby279 (AddGene #99130) to allow excitatory and inhibitory neurons to be identified by fluorescence at the time of recording.

Recordings were performed *via* conventional whole-cell current or voltage clamp methods at DIV13–16 using a Multiclamp 700B amplifier and a Digidata 1550 digital-to-analog converter. Signals were recorded at a 10-kHz sample rate and filtered at 3 KHz with a low-pass Bessel filter using pCLAMP 10 software (all equipment from Molecular Devices). Patch pipettes were fabricated with a P-97 pipette puller (Sutter Instruments) using 1.5 mm outer diameter and 1.28 mm inner diameter filamented capillary glass (World Precision Instruments). Pipette resistance was 3–6 MΩ. The external recording solution contained 145 mM NaCl, 5 mM KCl, 10 mM HEPES, 10 mM glucose, 2 mM CaCl_2_, and 2 mM MgCl_2_. The pH was adjusted to 7.3 using NaOH and the osmolality was adjusted to 325 mOsm with sucrose. The pipette solution contained 130 mM K^+^ methanesulphonate, 10 mM Na^+^ methanesulphonate, 1 mM CaCl_2_, 10 mM EGTA, 10 mM HEPES, 5 mM MgATP, and 0.5 mM Na_2_GTP (pH 7.3, 305 mOsm). The calculated free Ca^2+^ concentration (Schoenmakers et al., 1992) was about 22 nM. Experiments were performed at room temperature (21–23°C). A−14 mV liquid junction potential between the pipette and external solutions was calculated empirically, and the correction was applied before the experiment. Only cells with a stable series resistance of <15 MΩ were used for analysis.

Neuronal excitability was assessed using current clamp recording. For these experiments, resting membrane potential was measured immediately following the establishment of the whole-cell configuration. During recordings, current (<100 pA) was manually injected to hold the cells at approximately −60 mV. Membrane resistance and capacitance were calculated from the membrane potential changes in response to 1 s duration hyperpolarizing current steps that decreased in−5 pA increments. Action potentials were evoked and rheobase was obtained using 1 s duration depolarizing current steps that increased incrementally by 5–20 pA. An action potential was defined as a transient depolarization of the membrane which had a minimum rise rate of >10 mV/ms and reached a peak amplitude of >0 mV. Action potential characteristics were measured from the first action potential at rheobase. The threshold potential was measured at the point where the voltage increases at a rate >10 ms/mV. The duration was calculated from the full width at the half maximum voltage. For this calculation, the amplitude was measured from 0 mV to the peak potential. The maximum number of action potentials was measured from a 1-s current step.

Baclofen-evoked currents were recorded using the voltage clamp technique at a holding potential of−65 mV (corrected for junction potential). Series resistance was compensated at 60%. The external recording solution was supplemented with 300 nM tetrodotoxin, 25 μM bicuculline, 10 μM CNQX, and 100 μM AP-5 (all purchased from Hello Bio). Cells were perfused with an external recording solution at a rate of 0.25 mL/min. 0.1, 1.0, and 10 μM baclofen solutions were applied sequentially to neurons using a custom-built perfusion system with a 250-μm aperture located approximately 500 μm from the soma of the recorded cells. The solution exchange time at the recorded neuron was <0.5 s.

Quantification was carried out using custom-written scripts for Igor Pro v. 6 (Wavemetrics, USA) and R v. 3 (www.R-project.org). Statistical comparisons were made using the tests indicated in the Results section. *P*-values <0.05 were considered significant.

### DNA and RNA for oocyte expression

The DNAs encoding the proteins expressed in the oocytes, and RNA preparation, were as described (Reddy et al., 2021). GIRK1 (rat), GIRK2A (mouse), G*β*_1_ (bovine), and Gγ_2_ (bovine) cDNAs used in this study were cloned into high-expression oocyte vectors pGEM-HE or pGEM-HJ (Rishal et al., 2005). To express human GIRK2 (GeneBank: P48051), we used DNA synthesized on our request by ThermoFisher. To ensure robust activation by G*βγ*, the GIRK1/2 channels were expressed at a low surface density of ~3–5 channels/μm^2^ (Yakubovich et al., 2015) by using low doses of GIRK1 and GIRK2 RNAs (0.025–0.5 ng RNA/oocyte), which resulted in GIRK basal current (I_basal_) of ~2–3 μA and maximal G*βγ*-evoked currents of ~8–12 μA with saturating doses of G*βγ* for heteromeric GIRK1/2. In contrast, to obtain comparable surface levels and G*βγ*-evoked currents of homotetrameric GIRK2 or GIRK2-YFP (Kahanovitch et al., 2014), we had to inject 2 ng of GIRK2 RNA/oocyte. Note that I_basal_ for GIRK2 was very low, ~0.05–0.1 μA. The amounts of G*βγ* RNAs injected (per oocyte) were 5 ng G*β*_1_ and 2 ng Gγ_2_.

### Electrophysiology in oocytes

Oocyte defolliculation, incubation, and RNA injection were performed as described previously (Rishal et al., 2005; Rubinstein et al., 2009). The standard extracellular ND96 solution contained (in mM) 96 NaCl, 2 KCl, 1 MgCl_2_, 1 CaCl_2_, and 5 HEPES, and was titrated with NaOH to a pH of 7.6–7.8. Oocytes were defolliculated with collagenase (Type 1A, Sigma) in Ca^2+^-free ND96 solution and injected with 50 nL of RNA, and incubated for 2–4 days in NDE solution (ND96 solution supplemented with 2.5 mM pyruvate and 50 μg/ml gentamicin) at 20°C before testing. CaCl_2_ was omitted in Ca^2+^-free ND96. Whole-cell GIRK and Ca_v_3.2 currents in oocytes were measured using a two-electrode voltage clamp (TEVC) with Geneclamp 500 (Molecular Devices) and using agarose cushion electrodes filled with 3M KCl, with resistances of 0.1–0.8 MΩ for current electrode and 0.2–1.5 MΩ for voltage electrode. To measure GIRK currents, we used ND96 solution or high-K^+^ solution (HK24), in mM 24 KCl, 72 NaCl, 1 CaCl_2_, 1 MgCl_2_, and 5 HEPES. The pH of all solutions was 7.4–7.6.

ETX was dissolved in double-distilled water to obtain a 1-M stock solution. During the experimental procedure, we used eight concentrations of ETX solutions (in mM): 0.01, 0.03, 0.1, 0.3, 1, 3, 10, and 30. To prepare the experimental solutions, we diluted the ETX stock solution in HK24 to 30 mM, and the lower concentrations have been prepared by sequential dilutions.

The effect of ETX on GIRK was measured as follows: GIRK currents were measured at a holding potential of−80 mV while the solutions were changed from one to another. First, current was measured in ND96 solution for 5 s. Then, the solution was switched to HK24 for 30 s to measure the basal current. The next solution was HK24 with 0.01 mM ethosuximide for another 30 s, and the same step was repeated seven times for each of the ethosuximide solutions. Finally, HK24 + 2.5 mM Ba^2+^ solution was used to block all GIRK current and to reveal non-GIRK basal current.

### Analysis of dose–response relationships

Dose–response data were fitted to the different models using SigmaPlot 11 or 13 (Systat Software Inc.). The data were fitted to equations 1–3, representing three standard models: the standard one-component binding isotherm is


(1) $$\begin{array}{l} \left(\text{\% inhibition}\right)=100\text{x}/\left(\text{x}+{\text{K}}_{\text{d},\text{app}}\right), \end{array}$$

where x is drug concentration and K_d,app_ is the apparent dissociation constant; the one-site Hill equation is


(2) $$\begin{array}{l} \left(\text{\% inhibition}\right)=100{\text{x}}^{\text{n}}/\left({\text{x}}^{\text{n}}\;+{\text{K}}_{\text{d},\text{app}}^{\text{n}}\right), \end{array}$$

where n is Hill coefficient; and the two-component binding isotherm is


(3) $$\begin{array}{l} \left(\text{\% inhibition}\right)=100\text{cx}/\left(\text{x}+{\text{K}}_{\text{d}1,\text{app}}\right)\\ \;+100\text{x}\left(1-\text{c}\right)/\left(\text{x}+{\text{K}}_{\text{d}2,\text{app}}\right), \end{array}$$

where K_d1,app_ and K_d2,app_ are apparent dissociation constants for the 1st and 2nd binding sites, respectively, and c is the fraction of inhibition obtained by the binding of ETX to the high-affinity site.

### Imaging of G*βγ* in giant plasma membrane patches of *Xenopus* oocytes

Giant excised plasma membrane patches were prepared, stained with antibodies, and imaged as described (Peleg et al., 2002). Oocytes were mechanically devitellinized using fine forceps in a hypertonic solution (in mM: NaCl 6, KCl 150, MgCl_2_ 4, HEPES 10, and pH 7.6). The devitellinized oocytes were transferred onto a Thermanox™ coverslip (Nunc, Roskilde, Denmark) immersed in a Ca^2+^-free ND96 solution, with their animal pole facing the coverslip, for 10–20 min. The oocytes were then suctioned using a Pasteur pipette, leaving a giant membrane patch attached to the coverslip, with the cytosolic face toward the medium. The coverslip was washed thoroughly with fresh ND96 solution and fixed using 4% formaldehyde for 30 min. Fixed giant plasma membrane patches were immunostained in 5% milk in phosphate buffer solution (PBS). Non-specific binding was blocked with Donkey IgG 1:200 (Jackson ImmunoResearch #017-000-003). Anti-G*β* rabbit polyclonal antibody (T-20, Santa Cruz Biotechnology #SC-378) was applied at 1:200 dilution, for 45 min at 37°C. Anti-rabbit IgG DyLight 649-labeled secondary antibody (1:400; SeraCare Life Sciences #072-05-15-06) was then applied for 30 min at 37°C, washed with PBS, and mounted on a slide for visualization. Imaging was done with the LSM 510 META confocal microscope (Zeiss) with a 20× -air objective or a 40× -water immersion objective using the 633 nm laser in spectral mode, and emission was measured at 673 nm. Imaging of proteins in giant plasma membrane patches was performed using the confocal microscope in λ-mode. Images were centered on the edges of the membrane patches, so that background fluorescence from the coverslip could be seen and subtracted.

### Statistical analysis

Sample sizes and statistical tests are detailed in the figure legends. Data are presented as mean ± SEM. Statistical analysis was performed on raw data, except Figure 4A (see legend). Each dataset was tested for normality (Shapira–Wilk test) and equal variance (Brown–Forsyth test). Data that did not show normal distribution were analyzed using non-parametric methods. Statistical analyses for adult behavioral tests were performed using Prism (GraphPad). Two-group comparisons were performed using unpaired two-tailed *t*-test with correction using the Holm–Sidak method or one-way repeated measures ANOVA with Bonferroni correction. Multiple group comparisons were done using two-way repeated measures ANOVA followed by the Sidak test. The correlation between movement and SWD duration or inter-SWD interval was determined by a Pearson test. All other statistics were performed in R using a Mann–Whitney U-test with 1,000 or 10,000 permutations, as indicated in legends (see the MEA methods section for more details). For oocyte electrophysiology experiments, statistical analysis was performed on raw data with SigmaPlot 13 (Systat Software Inc.) or Prism (GraphPad). Two-group comparisons were performed using a two-tailed *t*-test, if the data passed the Shapiro–Wilk normality test and the equal variance test, otherwise, a Mann–Whitney rank sum test was used. Multiple group comparisons were done using one-way ANOVA on ranks followed by Dunn's test.

## Results

### Characterization of the *Gnb*1^*K*78*R*/+^ mouse model and G*β*_1_ expression

We generated an inbred mouse line carrying the first mutation identified in a patient, K78R, using CRISPR/Cas9. The K78R knockin mutation (c.233 A>G; p.K78R human NM_002074, mouse NM_008142) was verified by Sanger sequencing and PCR (Supplementary Figures 1A–C). As a model for GNB1 haploinsufficiency, we also obtained a knockout line with a 71-bp deletion (Del71) (Supplementary Figure 1D). Both *Gnb*1^Del71^ and *Gnb*1^K78R^ lines showed homozygous embryonic lethality (Supplementary Figure 1E). However, heterozygous *Gnb*1^Del71^ pups (*Gnb*1^Del71/+^) were viable, while heterozygous K78R pups (*Gnb*1^K78R/+^) showed reduced viability on an isogenic C57BL/6NJ (B6NJ) background. To minimize premature lethality that was observed as a result of deficient maternal behavior of B6NJ dams, we also generated a mixed background with FVB.129. Indeed, F1 hybrid (F1H) litters were generally larger and healthier, as observed by increased survival (Supplementary Figure 1E). We observed normal G*β*_1_ protein levels in postnatal day 0 (P0) *Gnb*1^K78R/+^ cortex and, as expected, a ~50% reduction of G*β*_1_ levels in *Gnb*1^Del71/+^ mice compared to WT (Supplementary Figure 1F). We also observed high G*β*_1_ membrane expression and low cytosolic expression in *Gnb*1^K78R/+^ mice (Supplementary Figure 1G). Taken together, these data demonstrate that the K78R mutation does not confer a null allele and that the protein is properly targeted to the membrane.

We also assessed G*β*_1_ expression in primary cortical neurons from WT and *Gnb*1^K78R/+^ pups. G*β*_1_ is expressed in both excitatory and inhibitory neurons (Supplementary Figures 2A–C), but is not expressed in astrocytes (Supplementary Figure 2D). G*β*_1_ preferentially localizes in the soma, near the plasma membrane, and is not present in the axon initial segment (Supplementary Figures 2E, E'). G*β*_1_ is expressed in both deep and upper layer cortical neurons (Supplementary Figure 2F). Immunocytochemistry did not detect distinct differences in G*β*_1_ expression levels and localization patterns in WT and *Gnb*1^K78R/+^ neurons (Supplementary Figure 2E' and data not shown); however, the resolution of this method is not sufficient to detect moderate changes in total or plasma membrane expression levels.

### *Gnb*1^*K*78*R*/+^ mice exhibit phenotypes relevant to understanding clinical features of *GNB1* Encephalopathy

**Figure 1:**
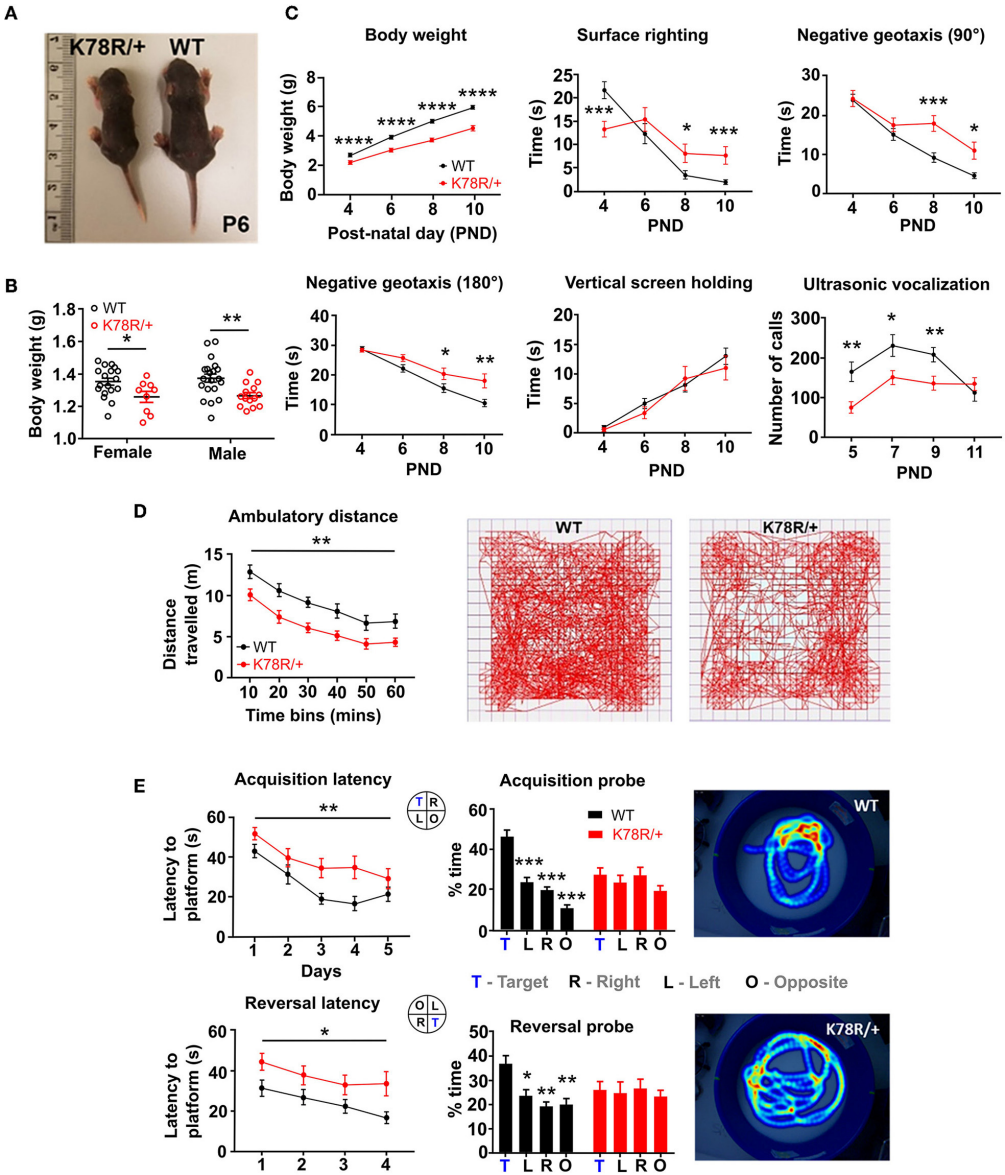
*Gnb*1^K78R/+^ mice exhibit developmental delay as neonates and motor and cognitive deficits as adults. **(A)** Representative photo of a *Gnb*1^K78R/+^ pup (left) and a WT littermate (right) at P6 on the B6NJ background. **(B)** Pup weight at P0 on the B6NJ background. WT (black circles): *n* = 18 female and 20 male mice; *Gnb*1^K78R/+^ (red circles): *n* = 9 female and 14 male mice; female *p < 0.05 vs. WT and male **p < 0.01 vs. WT. Mann–Whitney *U*-test with 10,000 permutations. **(C)** Developmental milestones between P4 and P10 on the B6NJ background include body weight, surface righting reflex, negative geotaxis 90° and 180° from the downward-facing position, and vertical screen holding: *n* = 32 WT and 20 *Gnb*1^K78R/+^. Separation-induced USV between P5 and P11: *n* = 28 WT and 20 *Gnb*1^K78R/+^. *p < 0.05; **p < 0.01; ***p < 0.001; ****p < 0.0001. Mann–Whitney U-test with 10,000 permutations. **(D)** Open field test on the B6NJ background. Distance traveled: *n*= 18 WT and 13 *Gnb*1^K78R/+^; **p < 0.01. Two-way repeated measures ANOVA. Representative movement traces of a WT (left) and a *Gnb*1^K78R/+^ (right) mouse walking for 1 h. **(E)** Morris water maze test on the B6NJ background. *n* = 15 WT and 14 *Gnb*1^K78R/+^. Learning: **p < 0.01 for acquisition latency; *p < 0.05 for reversal latency. Two-way repeated measures ANOVA. Memory: ***p < 0.001 for Target vs. Left, Target vs. Right, and Target vs. Opposite during the acquisition probe trial; *p < 0.05 for Target vs. Left, **p < 0.01 for Target vs. Right, and Target vs. Opposition during the reversal probe trial. One-way repeated measures ANOVA with Bonferroni correction. Representative heat maps of a WT (top) and a *Gnb*1^K78R/+^ mouse during the reversal probe trial. All graphs represent mean ± SEM. PND, postnatal day.

Both male and female *Gnb*1^K78R/+^ pups had a significantly lower body weight than WT littermates at birth (Figures 1A, B). To assess behavioral phenotypes pertinent to key clinical features of *GNB1* encephalopathy, we tested *Gnb*1^K78R/+^ pups for physical and sensorimotor developmental milestones between P4 and P11 and *Gnb*1^K78R/+^ adult mice for motor and cognitive functions. Early developmental milestones including body weight, surface righting reflex, negative geotaxis, vertical screen holding, and separation-induced ultrasonic vocalizations (USV) were assayed as previously described (Yang et al., 2012). We performed tests on both B6NJ and F1H backgrounds to assess phenotype stability. *Gnb*1^K78R/+^ pups weighed significantly less than WT littermates across early postnatal days (Figure 1C) and remained significantly smaller as young adults at 6 weeks (Supplementary Figures 3A, B). *Gnb*1^K78R/+^ pups exhibited deficits in the surface righting reflex test, being initially faster than WT littermates at P4 but not progressing afterward, and had a significant delay in both 90° and 180° negative geotaxis tests. They did not, however, show deficits in vertical screen holding, suggesting normal grip strength (Figure 1C). Finally, *Gnb*1^K78R/+^ pups emitted few vocalizations indicating abnormal development (Figure 1C). Pups on an F1H background were less extensively studied. As noted above, they appeared healthier than on a B6NJ background but presented similar, yet milder, developmental delays compared to WT littermates (Supplementary Figure 3B).

We assessed spontaneous locomotor activity in the open field, and observed that adult *Gnb*1^K78R/+^ mice were significantly less active compared to WT littermates (Figure 1D). Vertical exploration (rearing) and center time were not different (Supplementary Figure 3C), suggesting that reduced activity is not attributable to heightened anxiety-like behaviors in this test. *Gnb*1^K78R/+^ mice did not show deficits in the RotaRod test, suggesting normal balance and grip strength (Supplementary Figure 3D).

We tested learning and memory in adult mice. While *Gnb*1^K78R/+^ mice did not exhibit deficits in the classic contextual and cued fear conditioning tests (Supplementary Figure 3E), they displayed spatial learning and memory deficits in the Morris Water Maze test. These data suggest that, while *Gnb*1^K78R/+^ mice might recognize contextual cues, they are impaired in using spatial cues to navigate a complex environment. In the water maze, *Gnb*1^K78R/+^ mice were slower at acquiring the spatial learning task and the subsequent reversal task (Figure 1E). Importantly, these deficits were observed with normal swimming speed in *Gnb*1^K78R/+^ mice (Supplementary Figure 3F), indicating that swimming ability did not confound performance in this test. *Gnb*1^K78R/+^ mice also displayed memory deficits. During the probe test of both acquisition and reversal, WT mice spent significantly more time in the trained quadrant than in the other three quadrants, whereas *Gnb*1^K78R/+^ mice did not show a preference for the quadrant where the platform used to be (Figure 1E and Supplementary Figure 3G). In conclusion, behavioral phenotypes in *Gnb*1^K78R/+^ mice on the NJ background might be relevant to understanding *GNB1* encephalopathy, which includes global developmental delay, ambulatory deficits, and intellectual disability. More comprehensive behavioral analysis is needed to understand the roles of sex and genetic background in the manifestation of impairments.

### Excitability phenotypes in *Gnb*1^*K*78*R*/+^ mice

**Figure 2:**
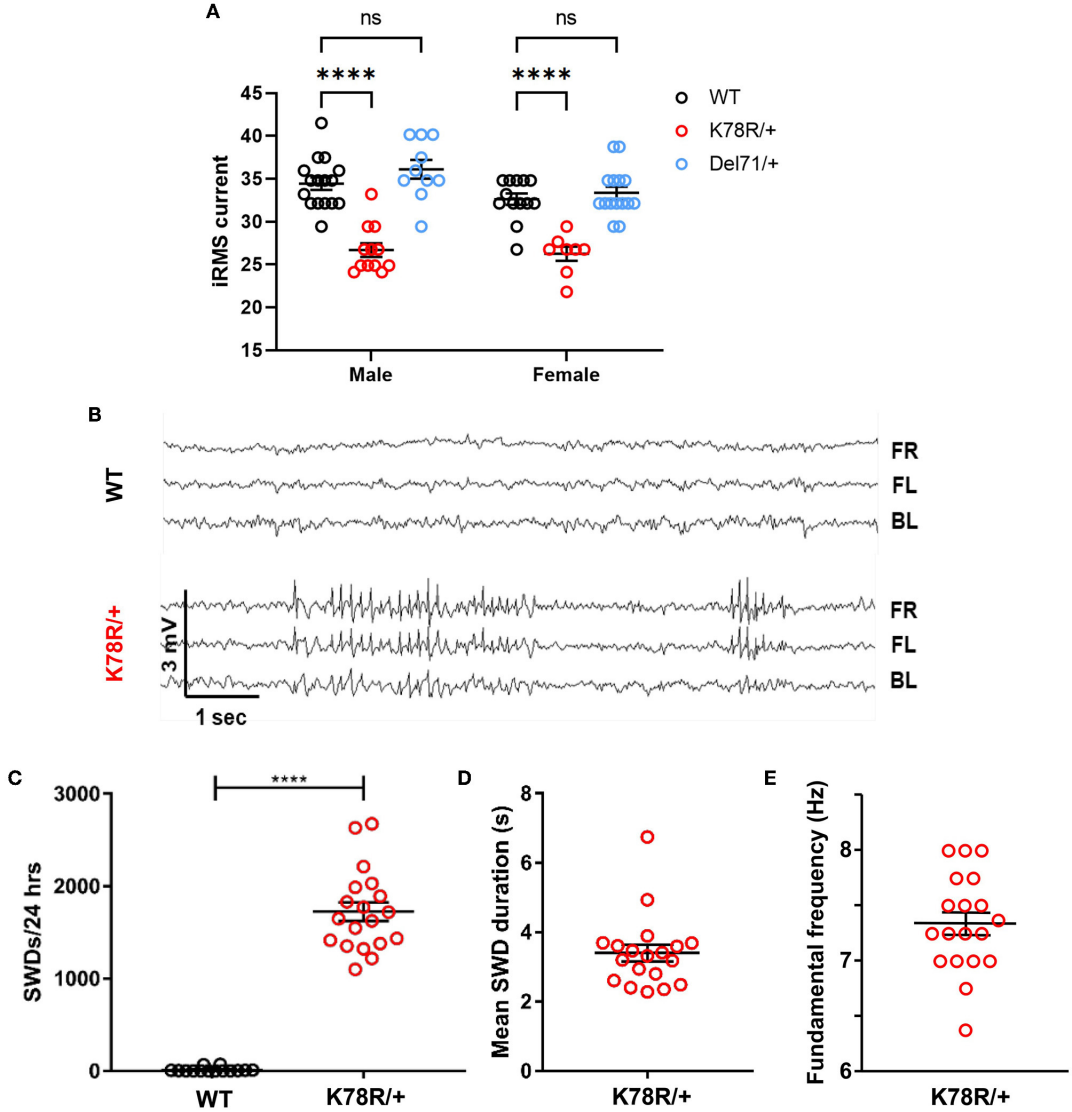
Excitability phenotypes in *Gnb*1^K78R/+^ mice. **(A)** Electroconvulsive threshold tests. Minimal clonic forebrain seizure endpoint in *Gnb*1^+/+^, *Gnb*1^K78R/+^, and *Gnb*1^Del71/+^ mice (WT *n* = 16 male, 13 female; *Gnb*1^K78R/+^ *n* = 12 male and 8 female; *Gnb*1^Del71/+^ n = 10 male, 15 female). **(B)** Representative traces from video-EEG recordings of cortically implanted *Gnb*1^K78R/+^ and WT littermates on the F1H background showing SWDs in *Gnb*1^K78R/+^ mice. FR, front right; FL, front left; BL, back left. **(C)** Quantification of SWDs per 24 h. *n* = 14 WT and 19 *Gnb*1^K78R/+^. WT = 11.71 ± 6.527 vs. *Gnb*1^K78R/+^ = 1,724 ± 100.6. **(D)** The mean duration of SWDs. *n* = 19 *Gnb*1^K78R/+^ mean ± SEM: 3.397 ± 0.239. **(E)** The fundamental frequency of peak spectral power. *n* = 19 *Gnb*1^K78R/+^ mean ± SEM: 7.342 ± 0.101. All graphs represent mean ± SEM. ****p < 0.0001 n.s., non-significant by two-way ANOVA with Dunnett's test for multiple comparisons (a) and Mann–Whitney *U*-test with 10,000 permutations (b).

Seizures, in a variety of types, including absence-type epilepsy, are common among patients with *GNB1* encephalopathy. We did not observe spontaneous convulsive seizures in *Gnb*1^K78R/+^ mice, and consequently assessed seizure susceptibility by electroconvulsive threshold (ECT) testing in *Gnb*1^K78R/+^ mice using stimulus settings for the minimal generalized (clonic forebrain) seizure endpoint compared to both *Gnb*1^+/+^ (WT) and *Gnb*1^Del71/+^. Female and male *Gnb*1^Del71/+^ mice showed ECT values equivalent to WT controls (Figure 2A), demonstrating that *Gnb1* haploinsufficiency does not lead to enhanced seizure susceptibility. *Gnb*1^K78R/+^ mice, however, showed a significantly lower seizure threshold compared to WT (Figure 2A), indicating that the K78R mutation may result in hyperexcitability.

We next used video-electroencephalogram (EEG) to examine *Gnb*1^K78R/+^ mice for spontaneous seizure events. *Gnb*1^K78R/+^ mice exhibited highly frequent generalized bilateral spike-and-wave discharges (SWDs) (Figures 2B, C; mean ± SEM *Gnb*1^K78R/+^: 1723 ± 108.9), while WT mice exhibited extremely rare SWDs (Figures 2B, C; mean ± SEM WT: 11.7 ± 6.5) in a 24 h period. The mean duration of SWDs ranged from 2.2 to 6.7 s (Figure 2D), with some events lasting longer than 30 s, and the fundamental frequency shows that the peak spectral power was 7.3 Hz (Figure 2E). We observed similarly frequent SWDs on both the F1H and B6NJ backgrounds. Together, these data show that *Gnb*1^K78R/+^ mice possess an enhanced susceptibility to seizures and display a high number of SWDs, an electrographic signature reminiscent of absence (blank-staring) seizures that are not convulsive, and usually do not affect the behaviors measured in standard tests such as those examined in this study (Frankel et al., 2005). This seizure type is often found in patients with *GNB1* encephalopathy (Petrovski et al., 2016; Hemati et al., 2018).

### *Gnb*1^*K*78*R*/+^ cortical networks display bursting phenotypes

**Figure 3:**
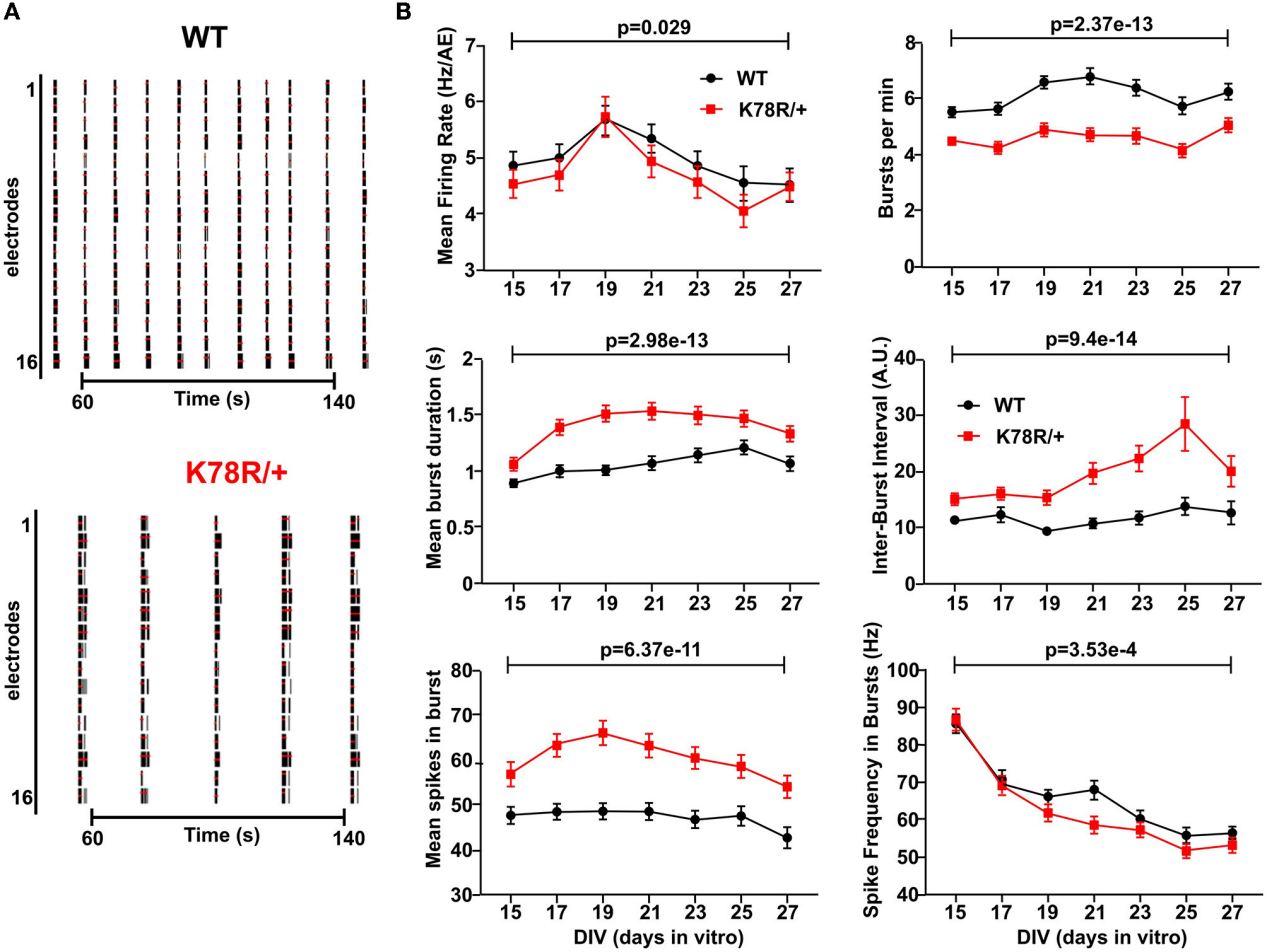
Excitability phenotypes in *Gnb*1^K78R/+^ cortical neurons in microelectrode arrays (MEA). **(A)** Raster plots showing WT and *Gnb*1^K78R/+^ firing across the 16 electrodes of a well of a representative plate, between 50 and 150 s of a 3-min recording at DIV36. The black bars indicate spikes, and the red bars indicate bursts. **(B)** Graphs representing spontaneous activity on MEA of *Gnb*1^K78R/+^ cortical neurons (red) and WT cortical neurons (black) from DIV15 to DIV27. Graphs show the combined analysis of nine plates from DIV15 to DIV27. *n* = 144 WT wells and 148 *Gnb*1^K78R/+^ wells from nine plates (8 biological replicates = 8 independent primary cultures). The combined MWU permutated p-values of each plate generated using the Fisher's method are shown.

Multi-electrode arrays (MEA) measure the local field potentials of cultured neurons within a larger neuronal network and are a popular means to assess neuronal network activity and synchrony. We used MEA to characterize the spontaneous activity of *ex vivo* neuronal networks from WT and *Gnb*1^K78R/+^ P0 cortices. Examination of MEA raster plots from WT and *Gnb*1^K78R/+^ networks (examples are shown in Figure 3A) revealed visible differences in bursting patterns. Using our custom MEA analysis package (Gelfman et al., 2018), we analyzed a variety of spiking and bursting features (see Methods). We show the combination of nine plates corresponding to eight biological replicates (Figure 3B). For each feature, we performed a Mann–Whitney U (MWU)-test followed by 1,000 permutations to compare activity in WT vs. mutant wells across the time points of interest. We also combined the permutated MWU *p*-values from each plate using the Fisher's method, which gave similar results (see Supplementary Table 1 for *p*-values from both statistical methods).

When comparing the activity (mean firing rate, MFR) of WT neurons with respect to time (days *in vitro*, DIVs), we observed an increase in MFR until DIV13, followed by stabilization of MFR. We thus present data from DIV15 to DIV27, when network activity has stabilized indicating functionally mature networks. Both WT and *Gnb*1^K78R/+^ networks developed at the same rate, as observed by similar increases in time to reach the number of active electrodes (nAE) per well, reaching a maximum by DIV15 (Supplementary Figure 4A). We observed striking bursting phenotypes in *Gnb*1^K78R/+^ networks relative to WT. Mutant neurons showed a decrease in burst frequency (decreased number of bursts per minute) as they fired very long bursts followed by very long inter-burst intervals (IBIs), leading to an increase in the mean number of spikes within a burst (Figures 3A, B). We observed only a small and delayed decrease in MFR, suggesting that MFR is not driving the bursting phenotype; however, there was a decreased spike frequency within bursts (Figure 3B).

Primary cultured neural networks become highly synchronized over time (Wagenaar et al., 2006; McSweeney et al., 2016). Therefore, we evaluated key synchrony parameters including the percentage of spikes in network spikes (NS) (Supplementary Figure 4B), which identifies highly synchronized short events (10 ms), as well as mutual information (Supplementary Figure 4C) and Spike Train Tiling Coefficient (Supplementary Figure 4D), which assess pairwise electrode correlations between nearby electrodes and at the well-level, respectively (Gelfman et al., 2018). For each feature, we show raw as well as normalized data recorded on each plate by dividing the activity in each well by the mean activity of WT wells. We did not observe significant differences between WT and *Gnb*1^K78R/+^ networks using any of the synchrony measures suggesting that altered synchrony does not underlie the bursting phenotypes of *Gnb*1^K78R/+^ networks.

### Ethosuximide corrects EEG and network firing patterns in the pre-clinical models

**Figure 4:**
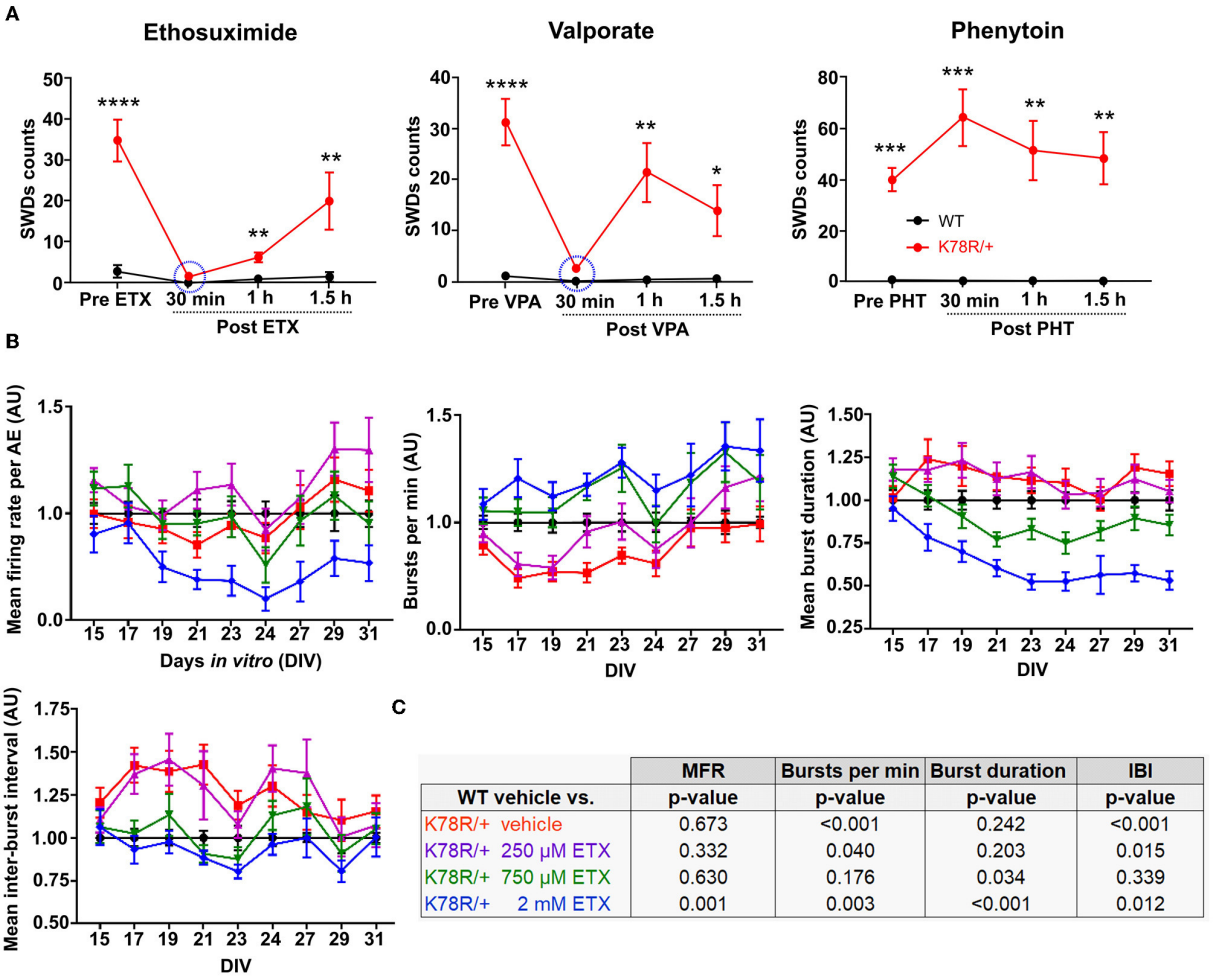
Pharmacological response of the pre-clinical models. **(A)** SWD manual counts on EEG recordings before and after acute treatment of adult mice with the AEDs ethosuximide (ETX; 200 mg/kg IP), valproate (VPA; 200 mg/kg IP), and phenytoin (PHT; 30 mg/kg IP). *n* = 17 WT and 21 *Gnb*1^K78R/+^ for ETX, 12 WT, and 21 *Gnb*1^K78R/+^ for VPA, and 5 WT and 7 *Gnb*1^K78R/+^ for PHT. IP, intra-peritoneal. For each time point, mean SWD counts have been compared using a t-test. *p < 0.05; **p < 0.01, ***p < 0.001; ****p < 0.0001. **(B)** Graphs representing spontaneous activity on MEA from DIV15 to DIV31 of untreated *Gnb*1^K78R/+^ cortical neurons (red), and *Gnb*1^K78R/+^ cortical neurons treated with 250 μM (purple), 750 μM (green) or 2 mM (blue) of ETX chronically from DIV3 to DIV31, normalized to WT cortical neurons (black). MFR, number of bursts per minute, mean burst duration, and mean IBI are shown from left to right. *n* = 30 wells from five biological replicates for all genotypes/conditions. **(C)** Statistics for the graphs shown in B. All *p*-values correspond to K78R/+ treatment compared to the WT vehicle using a Mann–Whitney *U*-test with 1,000 permutations. All graphs represent mean ± SEM. DIV, days *in vitro*; n.s., non-significant.

Ethosuximide (ETX) and valproic acid (VPA) are two antiepileptic drugs (AED) commonly used to treat absence seizures in humans, which also interrupt SWDs in rodent models (Marescaux et al., 1992; Manning et al., 2004; Vrielynck, 2013; Kim et al., 2015). Acute treatment of *Gnb*1^K78R/+^ mice with either ETX or VPA nearly abolished SWDs (Figure 4A). Owing to the short half-life of both drugs, SWDs progressively recovered an hour after treatment. Notably, while the ETX showed a short half-life in this mouse model, it is expected to be much longer in large mammals and humans (Patel et al., 1975; el Sayed et al., 1978). On the other hand, phenytoin, a voltage-gated Na^+^ channel blocker and an AED with a probable worsening effect on absence seizures in humans and rodents (Marescaux et al., 1992; Tokuda et al., 2007; Kim et al., 2015), did not affect SWD occurrence (Figure 4A).

We next investigated the effects of ETX on neural networks on MEA. Interestingly, even an acute application of ETX appeared to have a rescue effect on the aberrant bursting of *Gnb*1^K78R/+^ networks, decreasing the mean duration of bursts and IBIs (data not shown). Therefore, we wanted to determine whether chronic ETX exposure could have a lasting effect on the phenotype. ETX distributes equally among blood plasma, cerebrospinal fluid, and saliva (Löscher and Frey, 1984). The therapeutic range is typically between 300 and 750 μM (Patsalos et al., 2018), and can go as high as 1.1 mM (Kobayashi et al., 2009). We applied ETX on cultured networks at three different concentrations (250 μM, 750 μM, and 2 mM), every other day from DIV3 until DIV31. We observed that ETX reverted *Gnb*1^K78R/+^ aberrant bursting in a concentration-dependent manner (Figures 4B, C). At 250 μM, ETX did not affect the bursting phenotype, while at 750 μM, ETX did restore close-to-normal firing to the mutant network. ETX increased number of bursts per minute, decreased mean duration of bursts, and decreased IBIs, compared to the vehicle or 250 μM-treated *Gnb*1^K78R/+^ networks. Interestingly, 2 mM ETX seemed to produce an “overshoot” effect and greatly reduced the network activity. Overall, these data show that chronic ETX treatment, at therapeutic doses, leads to a cumulative and prolonged rescue of the *Gnb*1^K78R/+^ bursting phenotypes. The beneficial effects of ETX in *Gnb*1^K78R/+^ mouse and neuronal models implicate G*β*_1_ signaling in the mechanism of action of ETX.

### GABA_*B*_ receptor-evoked GIRK currents are larger in neurons from *Gnb*1^*K*78*R*/+^ mice

To determine whether the K78R mutation is associated with changes in intrinsic neuronal excitability, conventional whole-cell current clamp recording was performed on cultured cortical excitatory and inhibitory neurons obtained from WT and *Gnb*1^K78R/+^ mice. The neuronal cultures were transduced by viral vectors containing reporter genes under the control of the appropriate promoters (see Methods section), and the excitatory or inhibitory identity of the neurons was determined by fluorescence at the time of recording. As shown in Supplementary Table 2, and illustrated in Supplementary Figure 5, while there were small changes to the membrane resistance, capacitance, and action potential duration in *Gnb*1^K78R/+^ inhibitory neurons, there was no consistent pattern of changes to membrane characteristics that would indicate a clear change in basal excitability parameters.

**Figure 5:**
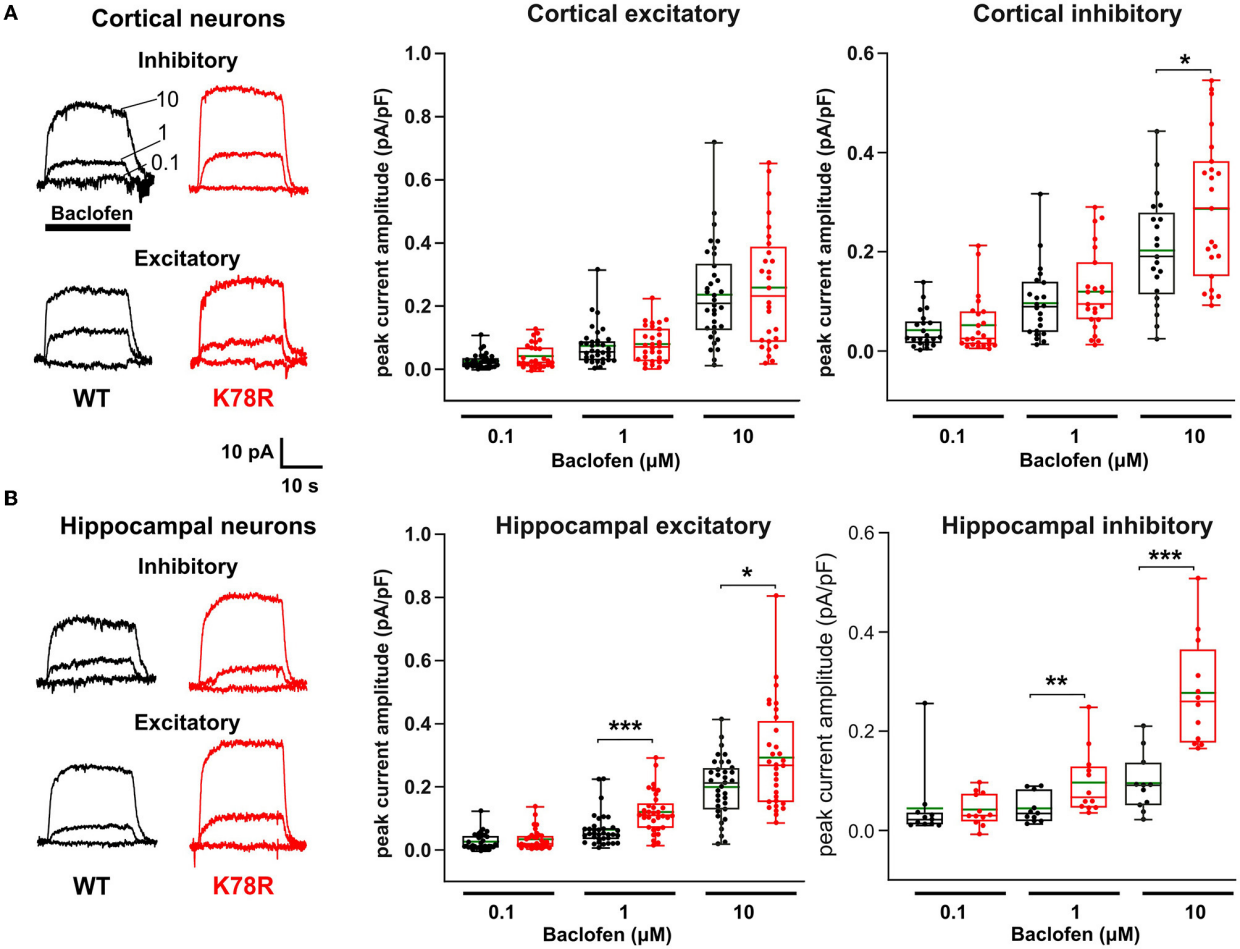
GABA_B_-evoked GIRK currents are larger in K78R/+ hippocampal and inhibitory cortical neurons. **(A)** Representative traces showing whole-cell voltage clamp recordings from inhibitory and excitatory WT (black) and K78R (red) cortical neurons. Overlaid traces recorded from individual neurons showing currents evoked by three 20 s sequential applications of increasing baclofen concentrations. Application period and concentrations are indicated on the top left recordings. Plots show the amplitudes of baclofen-evoked currents normalized to capacitance for each neuronal subtype, in single neurons. The box plot indicates the range, interquartile range, and median value; the green lines show mean values. Number of cortical excitatory neurons = 29 WT, 34 *Gnb*1^K78R/+^; number of cortical inhibitory neurons = 21 WT, 22 *Gnb*1^K78R/+^; from six litters. **(B)** Hippocampal neurons have the same layout as described above. Number of hippocampal excitatory neurons = 35 WT, 33 *Gnb*1^K78R/+^; number of hippocampal inhibitory neurons = 11 WT, 12 *Gnb*1^K78R/+^; from six litters. Comparisons between K78R and WT groups were performed using an unpaired *t*-test if the data passed the Shapiro–Wilk normality test or using the Mann–Whitney test if normality was not satisfied.

Given the prominence of GIRK channels as G*β*_1_ effectors that regulate the neuronal activity, we tested the hypothesis that there is aberrant activation of GIRK channels in neurons from *Gnb*1^K78R/+^ mice. Whole-cell voltage-clamp recording of GIRK currents was carried out following activation of GABA_B_ receptors using a 20-s baclofen application at 0.1, 1, and 10 μM. In neurons, baclofen-evoked outward currents are almost entirely mediated by GIRK channels (Luscher et al., 1997; Takigawa and Alzheimer, 1999; Koyrakh et al., 2005), with a half-maximal effect (EC_50_) at ~3 μM (Sodickson and Bean, 1996). Recordings were made from WT and *Gnb*1^K78R/+^ cultured excitatory and inhibitory neuronal subtypes obtained from both the cortex and the hippocampus. As shown in Figure 5A, the amplitude of baclofen-evoked current in cortical *Gnb*1^K78R/+^ inhibitory neurons was larger than in WT neurons at the highest (10 μM) baclofen concentration. Notably, no significant difference was observed in cortical excitatory neurons. Similarly, in hippocampal neurons (Figure 5B), the baclofen-evoked GIRK current was larger in both subtypes of *Gnb*1^K78R/+^ neurons with the most pronounced increase occurring in inhibitory neurons at the highest baclofen concentration. These results are consistent with the idea that changes in GIRK channel modulation contribute to the neuronal activity changes observed in *Gnb*1^K78R/+^ mice.

### K78R modulates GIRK1/2 and GIRK2 channels activation

**Figure 6:**
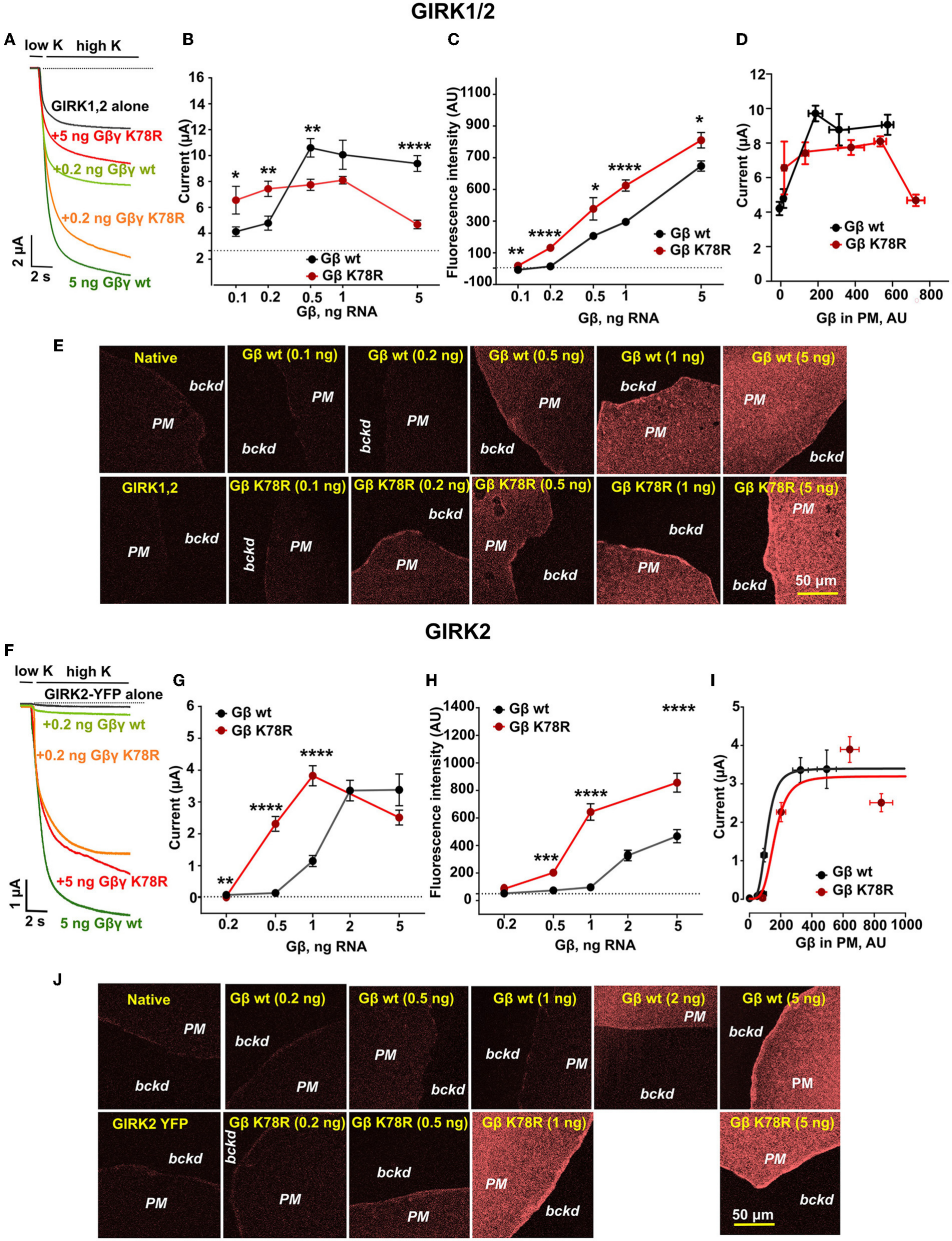
K78R is a GoF for GIRK1/2 and GIRK2 activation but, at high expression levels, shows an LoF for GIRK1/2. **(A)** Representative records of GIRK1/2 currents in *Xenopus* oocytes injected GIRK1 and GIRK2 RNAs (0.05 ng each), and the indicated amounts of G*β* RNA. The amount of Gγ RNA was 1/5 of G*β*. The dotted line shows zero current. **(B)** Summary of GIRK1/2 activation by G*β*WTγ and G*β*K78Rγ in the experiment shown in **(A)**, *n* = 8 to 13. The dotted line shows that the basal level of current in oocytes injected with GIRK1/2 RNA without coexpression of G*βγ*. At 0.1 ng G*β* RNA, there was an outlier point (~17 μA). The exclusion of the outlier did not greatly affect the statistical significance (*p* = 0.0243, *t* = 2.411, df = 23). **(C)** Changes in surface expression of G*β*WT and G*β*K78R as a function of RNA dose, in the presence of GIRK1/2, measured in giant excised membrane patches (GMP). Data shown are the net fluorescent signal produced by the expressed G*β*, after subtraction of the average signal observed in naïve (uninjected) oocytes, *n* = 8 to 18. **(D)** Dose-dependent activation of GIRK1/2 vs. actual surface expression of G*βγ* as measured in GMP. **(E)** Representative images of membrane patches at different G*β* RNA doses in the presence of GIRK1/2. **(F)** Representative records of GIRK2 currents in *Xenopus* oocytes injected with GIRK2 RNA (2 ng; C-terminally YFP-labeled GIRK2 was used) and the indicated amounts of G*β* RNA. The amount of Gγ RNA was 1/5 of G*β*. The dotted line shows zero current. **(G)** Summary of the experiment is shown in **(F)**, *n* = 6 to 14. **(H)** Changes in surface expression of G*β*WT and G*β*K78R as a function of RNA dose, in the presence of GIRK2, measured in GMP. Analysis as in **(C)**, *n* = 11 to 14. **(I)** Dose-dependent activation of GIRK2 vs. actual surface expression of G*βγ* as measured in GMP. The results were fitted to the Hill equation with a fixed Hill coefficient of 4. The fit yielded dissociation constant (Kd) of 118 and 162 AU and maximal currents of 3.4 and 3.18 μA for G*β*WTγ and G*β*K78Rγ, respectively. **(J)** Representative images of membrane patches at different G*β* RNA doses in the presence of GIRK2. All graphs represent mean ± SEM. For each G*β* dose, raw data for G*β*WT and G*β*K78R were compared using a two-tailed *t*-test or Mann–Whitney rank sum test. AU, arbitrary units.

To study the effects of the K78R mutation on GIRK channel activation by G*βγ*, GIRK1/2 or GIRK2 channel was expressed in *Xenopus* oocytes together with G*β*_WT_γ or G*β*_K78R_γ (Rubinstein et al., 2009). Exchange from physiological low-K^+^ ND96 to high-K^+^ HK24 solution resulted in the development of inward currents carried mostly by GIRKs (Figures 6A, F). GIRK1/2 and GIRK2 show substantial differences in gating: GIRK1/2 shows high, G*βγ*-dependent basal current (I_basal_) and a modest 1.5–4-fold activation by G*βγ* (Figure 6A; compare black and dark green traces), whereas GIRK2 has low, G*βγ*-independent I_basal_ and shows strong ~30–50-fold activation by G*βγ* (Rubinstein et al., 2009) (Figure 6F; compare black and dark green traces).

To capture the dose dependency of G*βγ* activation on GIRK channels, we titrated the amount of expressed G*βγ* protein by injecting increasing amounts of G*β*_WT_ or G*β*_K78R_ and Gγ RNA (5/1 RNA amounts, w/w). For GIRK1/2, small activation by G*β*_WT_γ was observed with 0.1 ng G*β* RNA, and saturation was reached at 0.5–1 ng G*β* RNA (Figure 6B and Supplementary Figure 6C). Interestingly, at low doses of G*β* RNA (0.05-0.2 ng), G*β*_K78R_γ activated GIRK1/2 better than G*β*_WT_γ (Figures 6A, B and Supplementary Figure 6C), suggesting gain-of-function (GoF). However, at higher G*β* doses, G*β*_K78R_γ ability to activate GIRK1/2 progressively decreased (Figure 6B and Supplementary Figure 6C). Notably, similar dose-dependent effects for G*βγ* were observed in the follow-up study with GIRK4 and GIRK1/4 (Reddy et al., 2021), which are homologous to GIRK2 and GIRK1/2, respectively.

To investigate whether the GoF at low G*βγ* levels and loss-of-function (LoF) at high G*β*_K78R_γ levels (i.e., RNA doses) are due to altered surface expression of G*β*_K78R_, we monitored the levels of membrane-attached G*βγ* in giant excised membrane patches of oocytes (Figure 6E) (Peleg et al., 2002; Rubinstein et al., 2009). We found that G*β*_K78R_γ consistently showed higher surface density than G*β*_WT_γ at all RNA doses (Figure 6C). To directly compare the actual dose-dependence of GIRK1/2 activation for G*β*_WT_γ and G*β*_K78R_γ, we plotted GIRK1/2 currents as a function of G*βγ* surface levels as measured in oocytes of the same groups (Figure 6D). Per equal amount of expressed G*βγ*, G*β*_K78R_γ activated GIRK1/2 similarly, or even less, compared to G*β*_WT_γ. The highest dose of G*β*_K78R_γ caused a large decrease in GIRK1/2 current, but we did not reach a similar expression level of G*β*_WT_γ to allow a direct comparison. The tendency for higher activation of GIRK1/2 at low doses of G*β*_K78R_γ RNA, and a smaller activation with high G*β*_K78R_γ doses, was consistently observed in additional experiments (Supplementary Figure 6C). Overall, these results suggest that K78R is a gain-of-expression mutation, and the apparent GoF is due exclusively to higher surface expression compared to G*β*_WT_γ. Further investigation of this phenomenon showed suppression of GIRK1/2 channel expression by high doses of G*β*_K78R_γ RNA and a reduction in channel open probability (Reddy et al., 2021), explaining the attenuated GIRK1/2 currents at high expression levels of G*β*_K78R_γ.

The activation of GIRK2 by coexpressed G*βγ* showed a similar pattern, with an apparent strong GoF for G*β*_K78R_γ at lower G*β* RNA doses which subsided at saturating RNA doses, 2–5 ng (Figures 6F, G, and Supplementary Figure 6C). With 0.5 ng G*β* RNA, GIRK2 current evoked by G*β*_K78R_γ was more than 6-fold larger than with G*β*_WT_γ (Figure 6G). Similar to GIRK1/2, the surface expression of G*β*_K78R_γ was higher than G*β*_WT_γ at all RNA doses (Figures 6H, J). Re-plotting GIRK2 activation as a function of G*βγ* surface levels showed that K78R activated GIRK2 similarly to G*β*_WT_γ in the range of surface expression studied (Figure 6I). Thus, similar to GIRK1/2, the physiological GoF of G*β*_K78R_γ on GIRK2 is due to gain of expression.

### Ethosuximide differentially inhibits GIRK1/2 and GIRK2 channels

**Figure 7:**
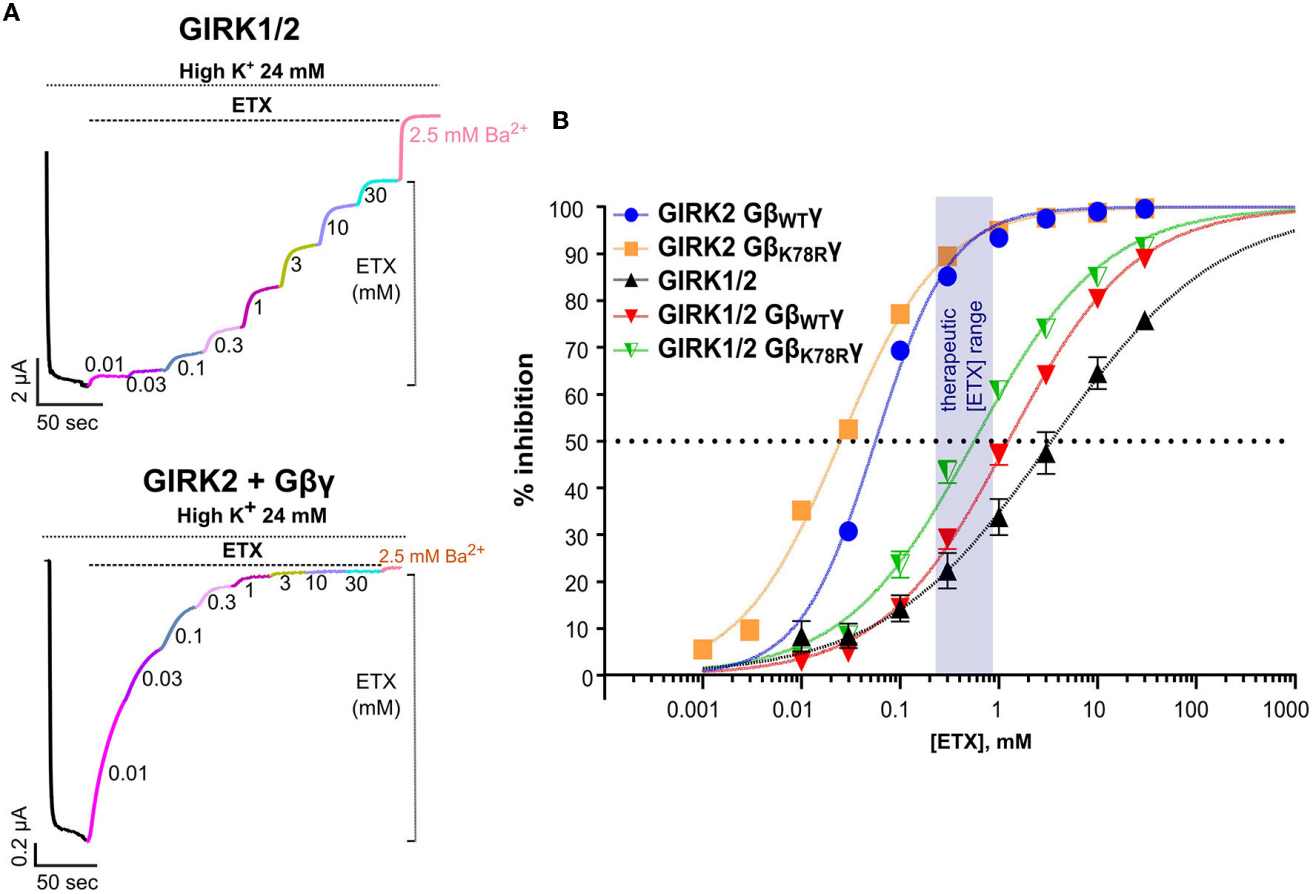
Ethosuximide blocks GIRK channels expressed in *Xenopus* oocytes. **(A)** Representative records showing the effect of ETX on I_basal_ of GIRK1/2 (top panel), and GIRK2 activated by coexpressed G*βγ* (bottom panel). Sequential application of increasing doses of the drug inhibited the current. Approximately 2.5 mM of Ba^2+^ was added at the end of the protocol for complete inhibition of GIRK. **(B)** Dose-dependence curves of ETX inhibition of GIRK1/2 and GIRK2 channels activated by saturating (5 ng RNA) doses of either G*β*_WT_γ or G*β*_K78R_γ and I_basal_ of GIRK1/2. The semitransparent rectangle indicates the therapeutic range of ETX concentrations. Each symbol is mean ± SEM, *n* = 6 to 19. The solid lines show fits to the Hill equation. Details of fits are shown in Supplementary Figure 5B. All graphs represent mean ± SEM.

We further tested the possible link between ETX-produced amelioration of the MEA bursting phenotypes and GIRK channels. The primary mode of action for ETX is thought to be through inhibition of low voltage-activated T-type Ca^2+^ channels (Ca_V_3) (see Discussion), but ETX has also been shown to inhibit GIRK channels *in vitro* (Kobayashi et al., 2009). We assessed ETX inhibition of heterotetrameric GIRK1/2 and homotetrameric GIRK2 in *Xenopus* oocytes (Figure 7 and Supplementary Figure 6). Both basal and G*βγ*-evoked GIRK1/2 currents were inhibited in a dose-dependent manner by ETX, with an EC_50_ of ~1.5–2 mM (Figures 7A, B and Supplementary Figures 6A, B), similar to a previous report (Kobayashi et al., 2009). I_basal_ of the mouse GIRK2, used throughout this study, was too small to reliably monitor inhibition. Using human GIRK2 that gave a larger I_basal_, we observed ETX inhibition with an EC_50_ of 3.7 ± 0.4 mM (*n* = 22), similar to the previous report (Kobayashi et al., 2009). Remarkably, G*βγ*-activated GIRK2 was inhibited at much lower doses (Figures 7A–C), with an EC_50_ of ~55 μM (Figure 7 and Supplementary Figures 6A, B). A milder increase in ETX potency by G*βγ* was also observed for GIRK1/2 (Figure 7B, Supplementary Figure 6B). Enhanced inhibition of GIRK by ETX following activation by G*βγ* is an interesting, and potentially clinically important, phenomenon deserving further study.

The dose–response curves for ETX inhibition of both GIRK1/2 and GIRK2 were well fitted by a Hill equation with apparent K_d_ (K_d,app_) values close to the observed EC_50_ (Figure 7 and Supplementary Figures 6A, B). However, for GIRK1/2, the low Hill coefficient (n_H_ ~0.5–0.6) could indicate two binding sites with different affinity—satisfactory fits were also obtained assuming two affinity components (Supplementary Figures 6A, B)—or two populations of channels with different sensitivity to block. Importantly, ETX inhibited both GIRK2 and GIRK1/2 channels activated by G*β*_K78R_γ with similar or even a slightly higher apparent affinity compared to G*β*_WT_γ (Figure 7B and Supplementary Figures 6A, B), in line with the strong effect of ETX on the *Gnb*1^K78R/+^ neurons.

## Discussion

We describe the first mouse model of *GNB1* encephalopathy carrying the pathogenic missense variant K78R. Prior studies emphasized the essential role of G*β*_1_ during brain development, as evidenced by the embryonic lethality of *Gnb1* knockout mice due to neural tube closure and neuronal progenitor cell proliferation defects (Okae and Iwakura, 2010). Here, we show that while the heterozygous K78R mutation does not impair brain development to the same degree as the knockout, it does affect neuronal activity by altering G*β*_1_ modulation of ion channels essential for the regulation of neuronal excitability.

*Gnb*1^K78R/+^ mice phenocopy many aspects of *GNB1* encephalopathy (Hemati et al., 2018), including developmental delay and motor and cognitive deficits (Figure 1), as well as abnormal neuronal excitability. *Gnb*1^K78R/+^ mice are not only more susceptible to generalized seizures but also have an extremely high incidence of absence-like seizures, clearly seen as SWDs in the EEG (Figure 2). This finding has been extended in a recent study (Teng et al., 2022) that capitalized on the *Gnb*1^K78R/+^ mouse model described here and published in a preprint form (Colombo et al., 2019). Teng et al. confirmed the presence of absence-like seizures in the EEG of *Gnb*1^K78R/+^ mouse and linked them to sensory regulation (Teng et al., 2022).

Consistent with the observed seizure susceptibility phenotypes, evaluation of mature neuronal network properties using MEA recordings of cultured cortical neurons revealed that *Gnb*1^K78R/+^ networks display hyperexcitability, firing long bursts that contain a greater number of spikes in each burst and longer periods between bursts (Figure 3). Activity-dependent network plasticity during early development is essential to establish precise connections for proper circuit activity including properties of network bursting, a process in which GABA signaling plays a central role (Ben-Ari, 2001; Cellot and Cherubini, 2013; Kirkby et al., 2013), while later in development, GABA acts to organize the ongoing temporal and spatial patterns of network activity (Trinchero et al., 2021). We reasoned that aberrant network bursting observed in *Gnb*1^K78R/+^ networks might, in part, result from an interaction between altered *GNB1* GPCR activity and metabotropic GABA receptor signaling, in particular, the activation of GIRK channels, given that GABA_B_ signaling requires functional G*βγ* (Luján et al., 2014). Indeed, with the GABA_B_ agonist baclofen at doses close to or higher than EC_50_, a gain of function (a greater GIRK response to baclofen) was evident in hippocampal neurons, and at 10 μM also in inhibitory cortical neurons (Figure 5).

In line with the increased GABA_B_-GIRK signaling in *Gnb*1^K78R/+^ neurons, we show in *Xenopus* oocytes that G*β*_1K78R_ exerts a GoF toward GIRK1/2 and GIRK2 activation at physiologically relevant mild G*β* RNA doses, owing to higher surface expression of G*β*_K78R_ compared with G*β*_WT_, in a wide range of G*βγ* expression levels (Figure 6). The gain-of-expression of G*β*_K78R_ was confirmed in our follow-up work, which also demonstrated that the K78R mutation does not affect the coupling of Gα_i/o_*βγ* to GPCRs and the G*βγ* regulation of voltage-gated Ca^2+^ channels, Ca_V_2.2 (Reddy et al., 2021), supporting the hypothesis that a defective G*βγ*-GIRK signaling plays an important role in the pathological neuronal function in K78R. However, in the case of GIRK1/2, there was a significant LoF toward GIRK1/2 at high G*βγ* doses, despite robust surface expression of G*β*_K78R_. This LoF was most probably due to a decrease in GIRK1/2 protein expression and open probability caused by overexpressed G*βγ* (Reddy et al., 2021). Mild levels of exogenously expressed G*βγ* mimic physiological conditions better than the high levels usually attained by saturating expression of a protein in heterologous cell models (Falkenburger et al., 2010; Yakubovich et al., 2015). This is supported by the observation that only a GoF was observed when GIRKs were activated in oocytes by G*β*_K78R_γ *via* a G_i/o_-coupled GPCR rather than by overexpressed G*βγ* (Reddy et al., 2021). Thus, our data obtained in the heterologous expression model meant to mimic physiological expression levels consistent with the data obtained in primary neurons. Therefore, we propose that, under physiological conditions, K78R is a GoF mutation for GIRK1/2 and GIRK2.

It is well established that SWDs result from hypersynchronized oscillations in cortico-thalamo-cortical circuits. These oscillations were commonly thought to originate from sustained burst firing of thalamic neurons (Danober et al., 1998; Huguenard and McCormick, 2007; Makinson et al., 2017), *via* increased Ca^2+^ currents through T-type voltage-gated calcium channels (Cheong and Shin, 2013; Cain et al., 2018). However, there is compelling evidence for a focal origin in specific regions of the somatosensory cortex (Meeren et al., 2002; Polack et al., 2007; Zheng et al., 2012; McCafferty et al., 2018). Interestingly, ETX can suppress SWDs originating in the cortical focus and restore normal neuronal activity (Manning et al., 2004; Polack and Charpier, 2009). We determined that VPA and ETX transiently abolished the SWDs in *Gnb*1^K78R/+^ mice. Furthermore, prolonged treatment with a therapeutic dose of ETX (0.75 mM) restored close-to-normal firing in the K78R mutant network in MEA. These findings raise the possibility that ETX acts, at least in part, through G*β*_1_ signaling which is affected by K78R. ETX is thought to act mainly as a T-type Ca^2+^ channel blocker (Coulter et al., 1989), although controversy remains as to the extent of this block at physiologically relevant doses of ETX (Gomora et al., 2001; Crunelli and Leresche, 2002; Tringham et al., 2012). However, there is no evidence yet supporting regulation of T-type channels by G*β*_1_ signaling—G*β*_2_, but not G*β*_1_, interacts with Ca_v_3.2 channels (Wolfe et al., 2003). ETX may reduce the Ca^2+^-activated K^+^ current and the persistent Na^+^ current (Leresche et al., 1998; Broicher et al., 2007). Notably, the Na^+^ channel blocker phenytoin did not suppress the SWDs in EEG (Figure 4A). Phenytoin blocks several types of voltage-gated Na^+^ channels with EC_50_ of 17–55 μM (Qiao et al., 2014) and also blocks T-type Ca^2+^ channels with an EC_50_ of 7–140 μM (Todorovic and Lingle, 1998; Todorovic et al., 2000; Lacinová, 2005). VPA acts on multiple targets, inhibiting histone deacetylases and Na^+^ and NMDA channels and enhancing GABA levels (Monti et al., 2009), but it does not inhibit GIRKs (Kobayashi et al., 2009) or T-type Ca^2+^ channels (Tringham et al., 2012). Together, these considerations support the hypothesis that, in countering GNB1 K78R-related SWDs, ETX acts on a target other than both Na^+^ channels and T-type Ca^2+^ channels.

Importantly, ETX was shown to inhibit basal GIRK currents in *Xenopus* oocytes (Kobayashi et al., 2009). We confirmed these findings and discovered that the apparent affinity of ETX is increased when the channels are activated by G*βγ* (Figure 7). Consequently, given the strong inhibitory activity of ETX on GIRK channels, in particular, the G*βγ*-activated GIRK2 homomers that show EC_50_ of ~50 μM (Figure 7), we hypothesize that one way in which ETX exerts its positive effects on network bursting and SWD control is through reductions in GIRK channel activity that is elevated as a consequence of the G*β*_K78R_γ being GoF for GIRKs. Homomeric GIRK2 channels are predominant in some midbrain nuclei (Lüscher and Slesinger, 2010; Luján and Aguado, 2015), but it is not clear how abundant they are in other brain structures: GIRK2 subunit is ubiquitously expressed throughout the brain, but it is usually co-localized with GIRK1 or GIRK3 subunits, and the proportion of GIRK2 homomers vs. GIRK1/2 or GIRK2/3 heterotetramers remains unknown. Nevertheless, the G*βγ*-activated GIRK1/2 (the predominant GIRK composition in the brain) shows EC_50_ of ~1 mM; thus, approximately 30–50% of G*βγ*-activated GIRK1/2 channels, and all G*βγ*-activated GIRK2 channels in neurons may be inhibited by the higher therapeutic doses of ETX (0.7–1.1 mM).

GIRK channels are essential regulators of neuronal excitability, mainly through neurotransmitter-induced postsynaptic prolonged IPSPs, and in some cases presynaptically, by inhibiting neurotransmitter release (Thompson et al., 1993; Lüscher and Slesinger, 2010; Luo et al., 2022). In many neuronal subtypes, GIRKs exhibit basal activity (Chen and Johnston, 2005; Wiser et al., 2006; Farhy Tselnicker et al., 2014) that regulates dendritic integration (Malik and Johnston, 2017) and, less prominently, intrinsic excitability [e.g., (Torrecilla et al., 2013; Prytkova et al., 2023), consistent with our results]. At the first glance, it may seem counterintuitive that blocking K^+^ (GIRK) channels would have an antiepileptic effect. However, there is evidence that the absence seizures can be enhanced by GABA_B_ signaling. In rodent models, activation of GABA_B_R exacerbates SWDs while inhibition, in either the thalamus or the cortex, suppresses SWDs (Hosford et al., 1992; Manning et al., 2004; Bortolato et al., 2010). Our electrophysiology results show preferential GIRK modulation in *Gnb*1^K78R/+^ inhibitory neurons. The ictogenic role of inhibitory interneurons has been demonstrated in several epilepsy models (Gnatkovsky et al., 2008; Yekhlef et al., 2015; Lévesque et al., 2022). We propose that one possible mechanism of GNB1^K78R/+^-induced epilepsy may be the increase in GIRK activity seen preferentially in inhibitory neurons (Figure 5). Enhanced basal or GABA_B_-induced GIRK activity would diminish the firing of inhibitory interneurons, leading to excessive, epileptogenic excitability of the network. Accordingly, disinhibition of these neurons by a GIRK blocker (here by ETX) would have an antiepileptic effect. An additional, or alternative, mechanism may operate *via* T-type Ca^2+^ channels, both in inhibitory and excitatory neurons. Indeed, it is thought that the GABA_B_R-mediated K^+^ slow IPSPs (generated by GIRK channels) de-inactivate the low threshold T-type Ca^2+^ current in thalamo-cortical cells, priming them for burst firing (Crunelli and Leresche, 1991), supporting the idea that increased GIRK signaling could underlie the bursting phenotype.

It is not clear how *GNB1* mutations that cause both GoF (K78R) and LoF (I80T/N) (Reddy et al., 2021) toward GIRK activation can cause epilepsy. Contributing factors could be a general disturbance in excitation–inhibition balance (Kaila et al., 2014) or differential localization (Luján and Aguado, 2015) and sensitivity to *GNB1* mutations (Reddy et al., 2021) of GIRKs of distinct subunit composition.

Our results indicate that the K78R mutation identified in individuals with *GNB1* encephalopathy affects cortical network activity and suggests that GoF of GIRK activation is an important part of the underlying mechanism. We provide evidence for a specific mode of action of ETX toward the G*β*_1_ signaling affected by the mutation in the cortex (i.e., inhibition of GIRK channels). Overall, we present the first animal model of *GNB1* encephalopathy, with several phenotypes relevant to clinical features and thereby representing a tool for translational research, and we implicate GIRK channels as an important component of how *GNB1* mutations cause disease.

## Data availability statement

The original contributions presented in the study are included in the article/Supplementary material, further inquiries can be directed to the corresponding author.

## Ethics statement

The animal study was reviewed and approved by Columbia Institutional Animal Care and Use Committee and by Tel Aviv University Institutional Animal Care and Use Committee.

## Author contributions

Conceptualization: SC, MB, HR, ND, and DG. Methodology: SC, BS, CM, YP, DW, and MB. Software: RD, SG, and YP. Formal analysis: SC, BS, HR, RD, SG, ER, YP, WF, and DG. Investigation: SC, SP, BS, HR, DW, and WF. Writing and reviewing: SC, MB, HR, MY, YP, CM, WF, ND, and DG. Visualization: SC, BS, HR, MB, and ND. Supervision: AB, CM, MY, YP, MB, WF, ND, and DG. Funding Acquisition: AB, ND, WF, and DG. All authors contributed to the article and approved the submitted version.
