# Supplementary material for: Epilepsy in a mouse model of GNB1 encephalopathy arises from altered potassium (GIRK) channel signaling and is alleviated by a GIRK inhibitor
Source: Front Cell Neurosci. 2023 May 18;17:1175895. doi: 10.3389/fncel.2023.1175895 (PMC10232839; doi:10.3389/fncel.2023.1175895)
Supplement: Supplementary file 1 [file Data_Sheet_1.PDF]

frontiers

Supplementary Material

Epilepsy in a mouse model of GNB1 Encephalopathy arises from altered potassium channel (GIRK) signaling and is alleviated by a GIRK inhibitor

Sophie Colombo[1#], Haritha P. Reddy[2,3], Sabrina Petri[1], Damian J. Williams[1], Boris Shalomov[2], Ryan S. Dhindsa[1], Sahar Gelfman[1], Daniel Krizay[1], Elizabeth E. Rafikian[4], Amal K. Bera[3], Mu Yang[1,4], Yueqing Peng[1,5], Christopher D. Makinson[1,5,6], Michael J. Boland[1,5] , Wayne N. Frankel[1,7], David B. Goldstein[1,7,&], Nathan Dascal[2,*]

[1] Institute for Genomic Medicine, Columbia University Irving Medical Center, New York, NY, USA
[2] Department of Physiology and Pharmacology, School of Medicine, Tel Aviv University, Tel Aviv, Israel
[3] Department of Biotechnology, Bhupat and Jyoti Mehta School of Biosciences, Indian Institute of Technology Madras, Chennai, India
[4] Mouse NeuroBehavior Core facility, Columbia University Irving Medical Center, New York, NY, USA
[5] Department of Neurology, Columbia University Irving Medical Center, New York, NY, USA
[6] Department of Neuroscience, Columbia University, New York, NY, USA
[7] Department of Genetics and Development, Columbia University Irving Medical Center, New York, NY, USA

S.C. current address – Dynacure, Illkirch-Graffenstaden, France
& D.B.G. current address - Actio Biosciences, San Diego, CA, USA

*Correspondence: N.D. dascaln@tauex.tau.ac.il

1 Supplementary Methods

1.1 Protein extraction from mouse cortices

P0 cortices were snap-frozen in liquid nitrogen and powdered using a mortar and pestle. Crude protein extracts were prepared by homogenizing the cortices in cold RIPA buffer (Sigma-Aldrich #R0278) containing a protease inhibitor cocktail (Thermo Fisher Scientific #A32955) and a phosphatase inhibitor cocktail (Sigma-Aldrich #4906837001). After vortexing for 1 min, the samples were incubated on a rotator for 15 min at 4 °C then centrifuged at 11000 rpm for 15 min at 4 °C to collect the supernatant.

For crude membrane/cytosol fractioning, cortices were homogenized in homogenization buffer (0.32 M sucrose, 10 mM HEPES pH 7.4, 2 mM EDTA in H_2O, containing a protease inhibitor and a phosphatase inhibitor cocktails). The samples were homogenized and centrifuged at 1000 g for 15 min at 4 °C to remove the pelleted nuclear fraction. The supernatant was further centrifuged at 16000 rpm for 20 min at 4 °C, yielding cytosolic fraction (supernatant) and membrane fraction (pellet). The pellet was resuspended in the homogenization buffer. Protein concentrations were determined with the Pierce BCA Protein Assay kit (Thermo Fisher Scientific #23225) using BSA as a standard.

1.2 Western blot

Sample proteins (10 µg) were mixed with 4x LDS buffer (Nupage), and 10x sample reducing agent (Nupage), heated for 10 min at 70 °C, separated on 4-12% Bis-Tris mini gel (Nupage) and transferred to a PVDF membrane (Millipore #ISEQ00010). Nonspecific binding was blocked for 1 h at room temperature with 5% blotto (Biorad) in Tris-buffered saline with 0.1% Tween (TBST). Membranes were incubated overnight at 4 °C with the primary antibody rabbit anti-GNB1 at 1/5000 (GeneTex #GTX114442), washed 3x 10 min with TBST and incubated with the secondary antibody HRP-conjugated goat anti-rabbit at 1/10000 (ThermoFisher Scientific #32260) in 5% blotto for 1 hour at RT and washed again 3x 10 min with TBST. Blots were incubated for 5 min with Pierce ECL Western Blotting substrate (Thermo Fisher Scientific #32106) and developed with a Kodak X-OMAT 2000A Processor. Blots were stripped for 15 min at RT in ReBlot Plus Strong solution (Millipore #2504), blocked for 30 min in 5% blotto, incubated for 1 h at RT with an HRP-conjugated mouse anti-β-actin antibody at 1/5000 (Santa Cruz Biotechnology #sc-47778) in 5% blotto and developed as previously described. β-actin was used as a loading control.

1.3 Immunocytochemistry

Primary cortical neurons were seeded on poly-D-lysine-coated 12 mm coverslips in 24-well plates at a density of 150,000 cells/well. At DIV14, cells were quickly washed with phosphate buffered saline (PBS) then fixed with 4% paraformaldehyde for 30 min at RT. Following three washes in PBS, cells were incubated with blocking solution (5% normal donkey serum, 1% bovine serum albumin, 0.3% Triton X-100 in PBS) for 1 hour at RT. Cells were incubated with primary antibodies diluted in blocking solution for 1.5 h at RT, washed three times with 0.2% Triton X-100 in PBS, then incubated with the secondary antibodies diluted in blocking solution for 30 min at RT in the dark. Finally, the cells were washed two times with 0.2% Triton X-100 in PBS, one time with PBS, and coverslips were mounted on SuperFrost microscopy slides with one drop of Prolong Diamond Antifade Mountant with DAPI (ThermoFisher Scientific #P36971) and allowed to cure overnight at RT in the dark before imaging.

Primary antibodies: rabbit anti-GNB1 at 1/200 (GeneTex #GTX114442), mouse anti-MAP2 at 1/500 (Sigma #M4403), mouse anti-GFAP at 1/300 (Cell Signaling Technology #3670), mouse anti-VGlut1 at 1/100 (Synaptic Systems #135511), mouse anti-Gad67 at 1/500 (EMD #MAB5406), mouse anti-Satb2 at 1/100 (Abcam #ab51502), rat anti-Ctip2 at 1/125 (Abcam #ab18465), rabbit anti-Tbr1 at 1/100 (Abcam #ab31940), mouse anti-Ankyrin G at 1/100 (Santa Cruz Biotechnology #sc-12719). Secondary antibodies: Alexa Fluor 488, 568 or 647 conjugated donkey anti-mouse, donkey anti-rabbit or goat anti-rat were used at 1/500 (ThermoFisher Scientific). Imaging was performed with an inverted AxioObserver Z1 epifluorescent microscope (Zeiss) equipped with an Axiocam 503 mono camera with a 20x air objective or a 40x oil immersion objective, and images acquired with the Zen 2 software. Post-processing was performed with Zen 2 (Zeiss) and ImageJ software. Only minimal adjustments to brightness and contrast were performed.

1.4 Adult learning and memory tests

Water maze acquisition and reversal: Spatial learning and reversal learning were assessed in the Morris water maze using procedures and equipment as described (Yang et al., 2012). The apparatus was a circular pool (120 cm diameter) filled 45 cm deep with tap water rendered opaque with the addition of nontoxic white paint (Crayola). Proximal cues were two stickers taped on the inner surface of the pool, approximately 20 cm above the water surface. Trials were videotaped and scored with Ethovision XT 12 (Noldus). Acquisition training consisted of four trials a day for 5 days. Each training trial began by lowering the mouse into the water close to the pool edge, in a quadrant that was either right of, left of, or opposite to, the target quadrant containing the platform. The start location for each trial was alternated in a semi-random order for each mouse. The hidden platform remained in the same quadrant for all trials during acquisition training for a given mouse, but varied across subject mice. Mice were allowed a maximum of 60 s to reach the platform. A mouse that failed to reach the platform in 60 s was guided to the platform by the experimenter. Mice were left on the platform for 15 s before being removed. After each trial, the subject was placed in a cage lined with absorbent paper towels and allowed to rest under an infrared heating lamp for 60 s. Acquisition training continued for 5 d, or until the WT control group reached criterion. Three hours after the completion of last training on day 5, the platform was removed and mice were tested in a 60 s probe trial. Parameters recorded during training days were latency to reach the platform, total distance traveled, and swim speed. Time spent in each quadrant and number of crossings over the trained platform location and over analogous locations in the other quadrants was used to analyze probe trial performance. Reversal training began 3 d after the completion of acquisition training. In reversal training trials, the hidden platform was moved to the quadrant opposite to its location during acquisition training, for each mouse. Procedures for reversal training and probe trial were the same as in the initial acquisition phase.

2 Supplementary Figures

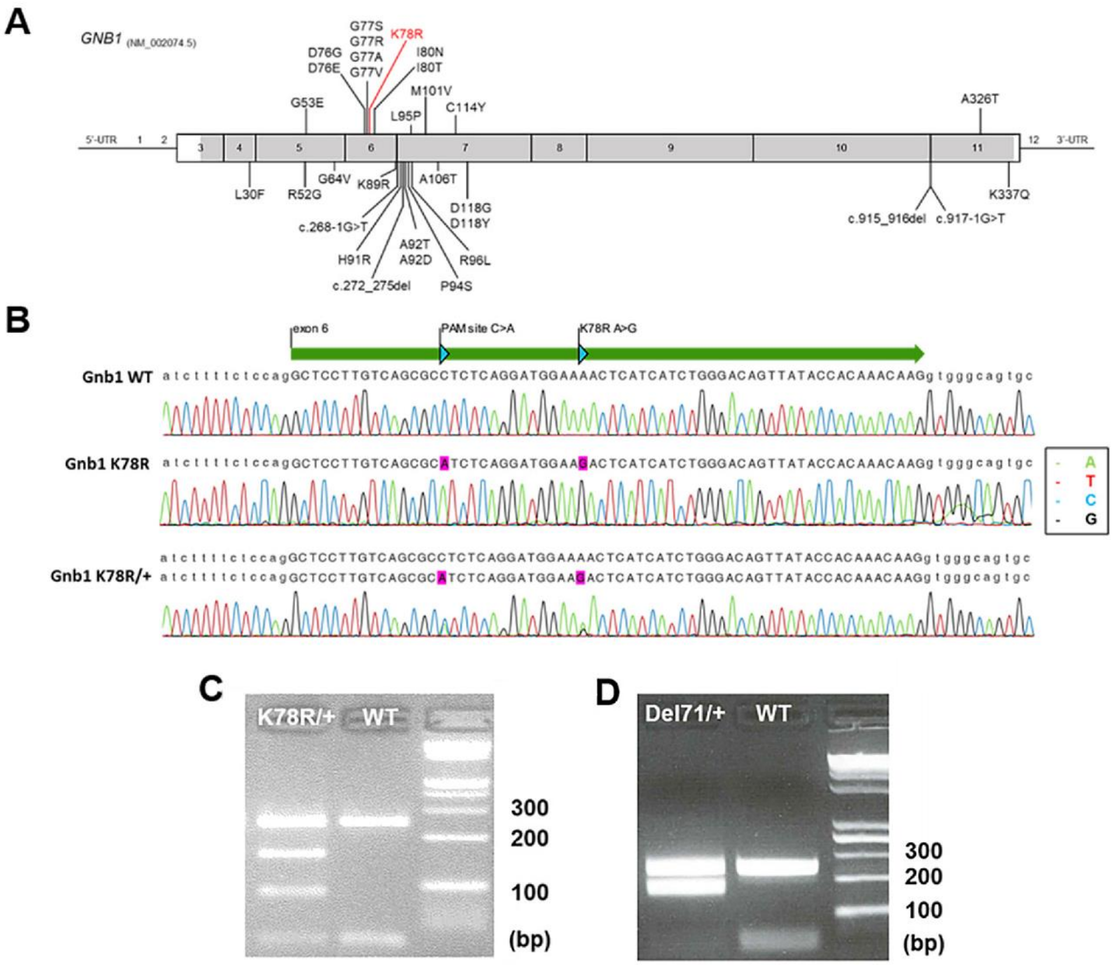

E

	K78R (B6NJ)					
	WT x Het (P0)		WT x Het (adults)		Het x Het (adults)	
	Oberved % (N)	Expected %	Oberved % (N)	Expected %	Oberved % (N)	Expected %
WT	63% (34)	50%	79% (129)	50%	57% (8)	25%
Het	37% (20)	50%	21% (35)	50%	43% (6)	50%
Hom	N/A	N/A	N/A	N/A	0% (0)	25%
Total	100% (54)	100%	100% (164)	100%	100% (14)	100%

	K78R (F1H)			
	WT x Het (adults)		Het x Het (adults)	
	Oberved % (N)	Expected %	Oberved % (N)	Expected %
WT	56% (79)	50%	54% (7)	25%
Het	44% (62)	50%	46% (6)	50%
Hom	N/A	N/A	0% (0)	25%
Total	100% (141)	100%	100% (13)	100%

	Del71 (B6NJ)			
	WT x Het (adults)		Het x Het (adults)	
	Oberved % (N)	Expected %	Oberved % (N)	Expected %
WT	46% (31)	50%	37% (10)	25%
Het	54% (37)	50%	63% (17)	50%
Hom	N/A	N/A	0% (0)	25%
Total	100% (68)	100%	100% (27)	100%

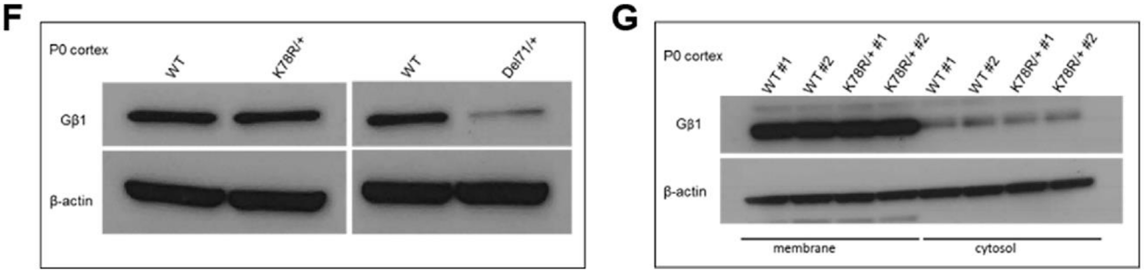

Supplementary Fig. 1. Generation and characterization of the K78R knock-in mouse model using CRISPR/Cas9. **(A)** Schematic of the *GNB1* coding sequence showing the position of published mutations. The K78R mutation located in exon 6 is highlighted in red. **(B)** Sanger sequencing chromatograms showing the WT (top) and K78R (middle) alleles obtained from individual bacterial clones following TOPO TA cloning of the PCR product obtained after amplification of the genomic DNA of a *Gnb1*$^{K78R/+}$ mouse. The third chromatogram corresponds to the direct sequencing of the PCR product, showing the overlay of WT and K78R alleles. **(C)** Typical genotyping result obtained by PCR followed by restriction digest with the *Hinf*I enzyme of tail DNA from a WT and a *Gnb1*$^{K78R/+}$ mouse. **(D)** Typical genotyping result obtained by PCR of tail DNA from a WT and a *Gnb1*$^{Del71/+}$ mouse. **(E)** Numbers and associated percentages of mice of each expected genotypes born from *Gnb1*$^{+/+}$ x *Gnb1*$^{K78R/+}$ or *Gnb1*$^{K78R/+}$ x *Gnb1*$^{K78R/+}$ mating at P0 and after weaning on the B6NJ and F1H backgrounds and from *Gnb1*$^{+/+}$ x *Gnb1*$^{Del71/+}$ or *Gnb1*$^{Del71/+}$ x *Gnb1*$^{Del71/+}$ mating on the B6NJ background. **(F)** Western Blot showing Gβ1 expression in WT, *Gnb1*$^{K78R/+}$ and *Gnb1*$^{Del71/+}$ P0 cortex. β-actin serves as a loading control. Knock-out *Gnb1*$^{Del71/+}$ mice show reduced expression compared to WT mice, as expected, but there is normal Gβ1 expression in *Gnb1*$^{K78R/+}$ mice, suggesting the K78R mutation does not lead to haploinsufficiency. **(G)** Western blot showing Gβ1 expression in two WT and two *Gnb1*$^{K78R/+}$ P0 cortices following membrane and cytosol fractioning. Most Gβ1 protein is localized in the membrane fraction in both WT and *Gnb1*$^{K78R/+}$ mice.

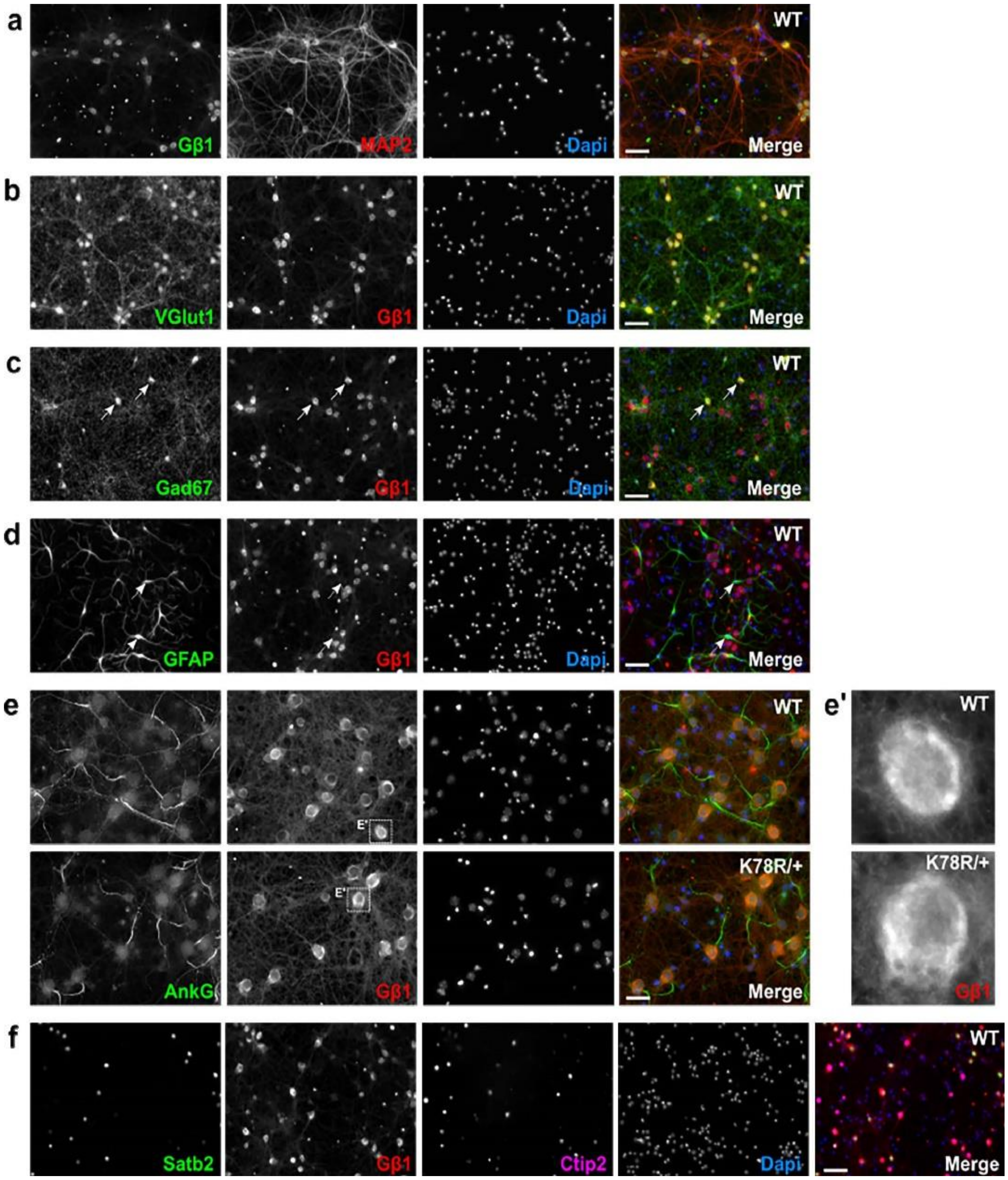

Supplementary Fig. 2. Gβ1 is strongly localized to the soma of both excitatory and inhibitory neurons. Immunocytochemistry of primary cortical neurons at DIV14. **a** Gβ1 (green) is strongly expressed in the soma of all neurons stained by MAP2 (red). **b** Gβ1 (red) colocalizes with the excitatory neuron marker, glutamate transporter VGlut1 (green). **c** Gβ1 (red) colocalizes with the inhibitory neuron marker, glutamate decarboxylase Gad67 (green). White arrows indicate example of cells showing colocalization. **d** Gβ1 (red) does not colocalize with the astrocyte marker GFAP (green). Dashed white arrows indicate example of cells with absence of colocalization. **e** Gβ1 (red) is not present in the axon initial segment, visualized with Ankyrin G (green) in both WT and *Gnb1*$^{K78R/+}$ neurons. **e'** Enlargement of a Gβ1+ neuronal soma from E. **f** Gβ1 (red) colocalizes with the deep layer marker Ctip2 (purple) and the upper layer marker Satb2 (green). Scale bars: 50 µm for a-d and f; 25 µm for e.

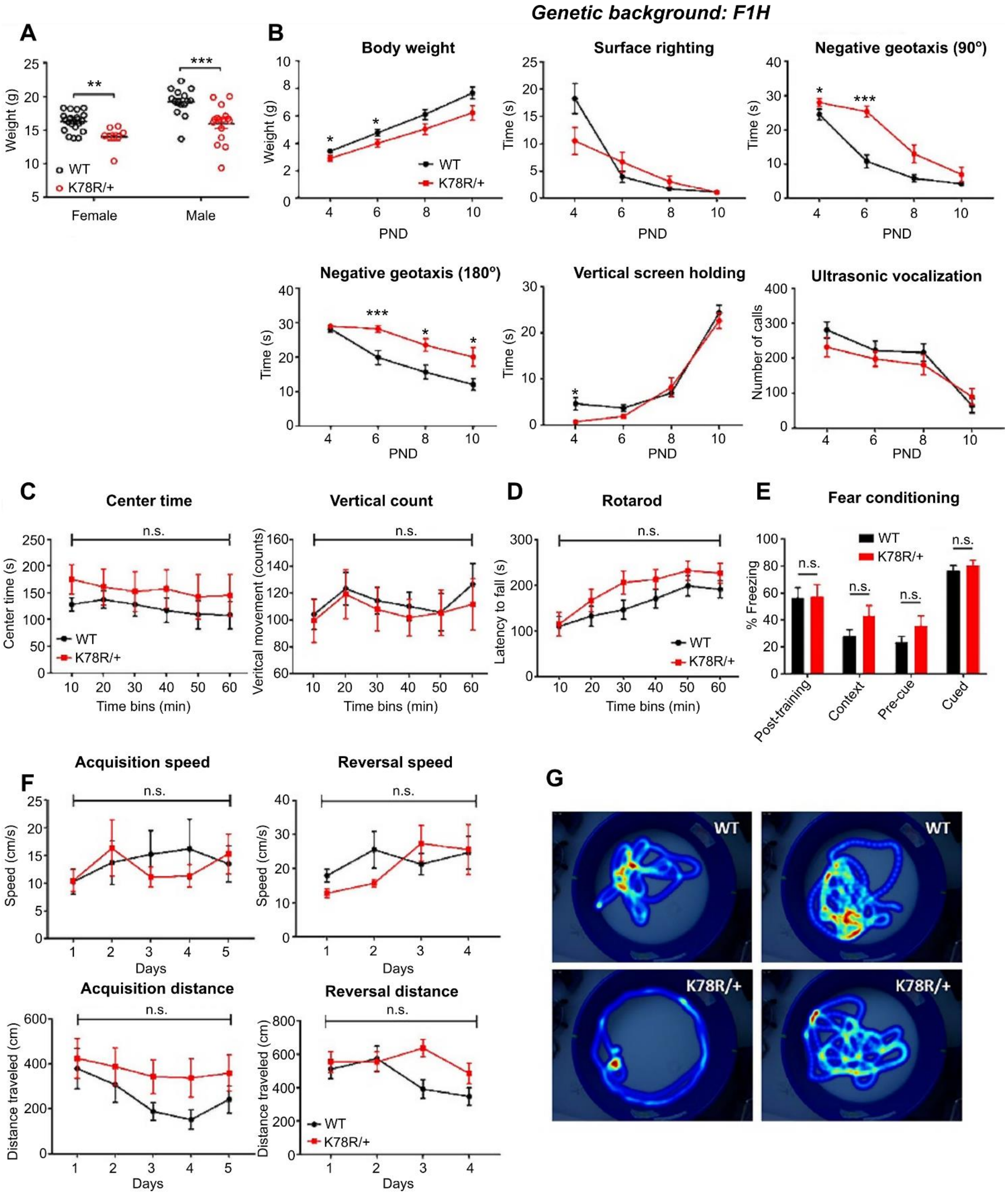

Supplementary Fig. 3 *Gnb1*$^{K78R/+}$ mice present with developmental delay, and motor and cognitive deficits. All data was collected on the B6NJ background except (b), which was collected on F1 hybrids. **(A)** Weight at 6 weeks old on the B6NJ background. WT (black circles): n = 21 females and 15 males; *Gnb1*$^{K78R/+}$ (red circles): n = 8 females and 17 males; females $^{**}p < 0.01$ vs. WT and males $^{***}p < 0.001$ vs. WT. Mann-Whitney U test

with 10,000 permutations. **(B)** Developmental milestones between P4 and P10 on the F1H background include body weight, surface righting reflex, negative geotaxis 90° and 180° from downward-facing position, and vertical screen holding: n = 16 WT and 15 *Gnb1*$^{K78R/+}$. Separation-induced USV between P5 and P11: n = 16 WT and 15 *Gnb1*$^{K78R/+}$. Note that we did not observe the typical inverted U-shaped curve for the WT pups, suggesting that USV may develop earlier on the F1H background. *$p < 0.05$; ***$p < 0.001$. Mann-Whitney U test with 10,000 permutations**.** **(C)** Additional parameters for open field on the B6NJ background: Vertical exploration and center time. n= 18 WT and 13 *Gnb1*$^{K78R/+}$; n.s.; Two-way repeated measures ANOVA. **(D)** Latency to fall in RotaRod test on the B6NJ background. n= 14 WT and 12 *Gnb1*$^{K78R/+}$; n.s.; Two-way repeated measures ANOVA. **(E)** Fear conditioning test for cued and contextual memory. n= 14 WT and 11 *Gnb1*$^{K78R/+}$; n.s.; Unpaired two-tailed t-test with Holm-Sidak correction. **(F)** Control parameters for the Morris Water Maze test on the B6NJ background. Both acquisition and reversal swim speeds and swim distances were unaffected. n = 15 WT and 14 *Gnb1*$^{K78R/+}$; n.s.; Two-way repeated measures ANOVA. **(G)** Additional representative heat maps from the reversal probe trial of the Morris Water Maze test showing a robust difference in search strategy between WT and *Gnb1*$^{K78R/+}$ mice. All graphs represent mean ± SEM. n.s., non-significant.

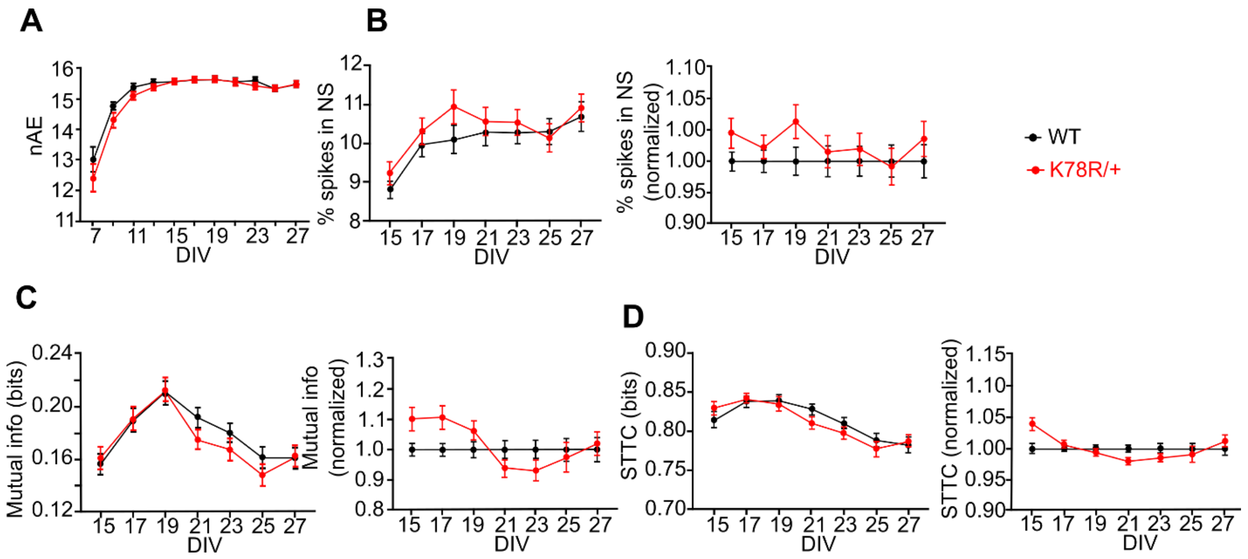

Supplementary Fig. 4. Excitability phenotypes in *Gnb1*$^{K78R/+}$ cortical neurons recorded on MEA. **(A)** Graph showing the number of active electrodes (nAE) per well for *Gnb1*$^{K78R/+}$ (red) and WT (black) from DIV7 to DIV27. n = 144 WT wells and 148 *Gnb1*$^{K78R/+}$ wells from 9 plates (8 biological replicates). The combined MWU permutated p-values of each individual plate using Fisher's method is shown. **(B-D)** Graphs representing spontaneous synchrony features on MEA. For each parameter, the left panel shows raw data, the right panel shows data for *Gnb1*$^{K78R/+}$ (red) normalized to WT (black) cortical neurons from DIV15 to DIV27. n = 144 WT wells and 148 *Gnb1*$^{K78R/+}$ wells from 9 plates (8 biological replicates). Permutated p-values were calculated with a Mann-Whitney U test followed by 1,000 permutations. **(B)** Percentage of spikes in network spikes (NS). **(C)** Mutual info. **(D)** Spike Train Tiling Coefficient (STTC).

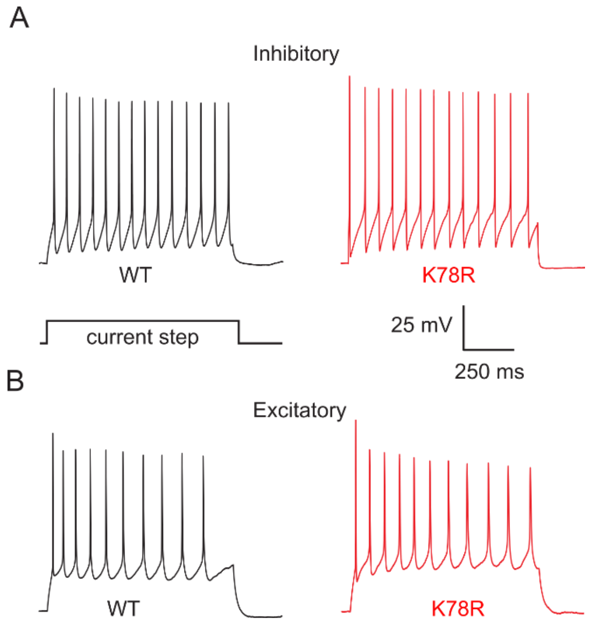

Supplementary Fig. 5. Action potential firing in inhibitory and excitatory cortical neurons from WT and *Gnb1*$^{K78R/+}$ mice. Representative current clamp recordings taken from neurons in which a 1 s duration current step was injected to elicit a train of action potentials. WT neurons are shown in black, K78R in red. For the traces shown here, the current step amplitude was that which evoked half the maximum number of action potentials (and varied between cells). The neurons expressed subtype-specific reporters which allowed inhibitory (A) and excitatory neurons (B) to be identified by fluorescence.

A

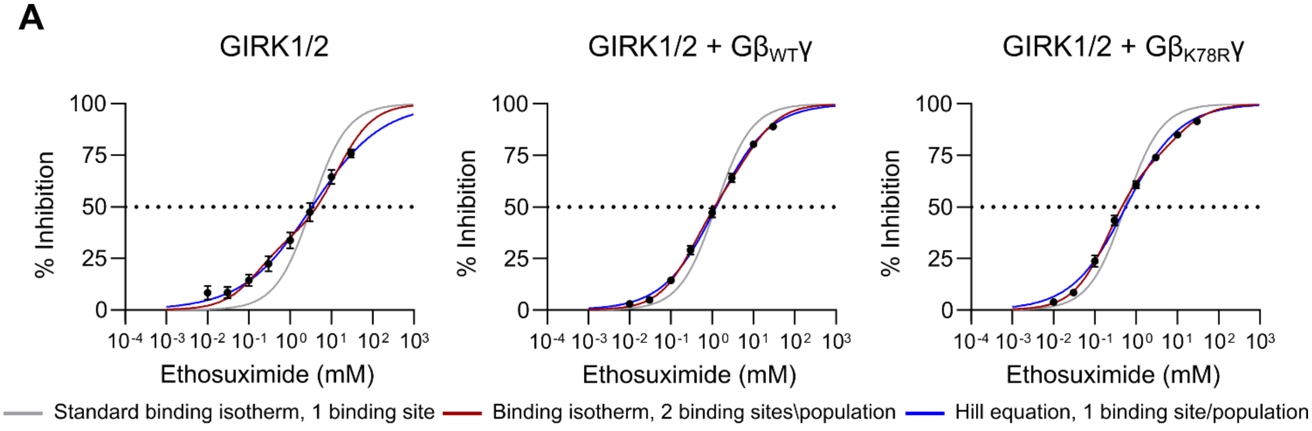

B

	One-site model	Hill model		Two-site model		
Channel	$K_{d,app}$ (mM)	$K_{d,app}$ (mM)	nH	$K_{d,app}$ site 1 (mM)	$K_{d,app}$ site 2 (mM)	fraction of high-affinity inhibition
GIRK2 + Gβγ$_{WT}$	0.055	0.056	1.14	0.054	NA	0.998
GIRK2 + Gβγ$_{K78R}$	0.0255	0.025	0.85	0.013	0.09082	0.35
GIRK1/2	3.3	3.4	0.51	0.17	14.15	0.36
GIRK1/2 + Gβγ$_{WT}$	1.2	1.24	0.68	0.32	7.779	0.57
GIRK1/2 + Gβγ$_{K78R}$	0.54	0.56	0.66	0.21	9.829	0.71

C

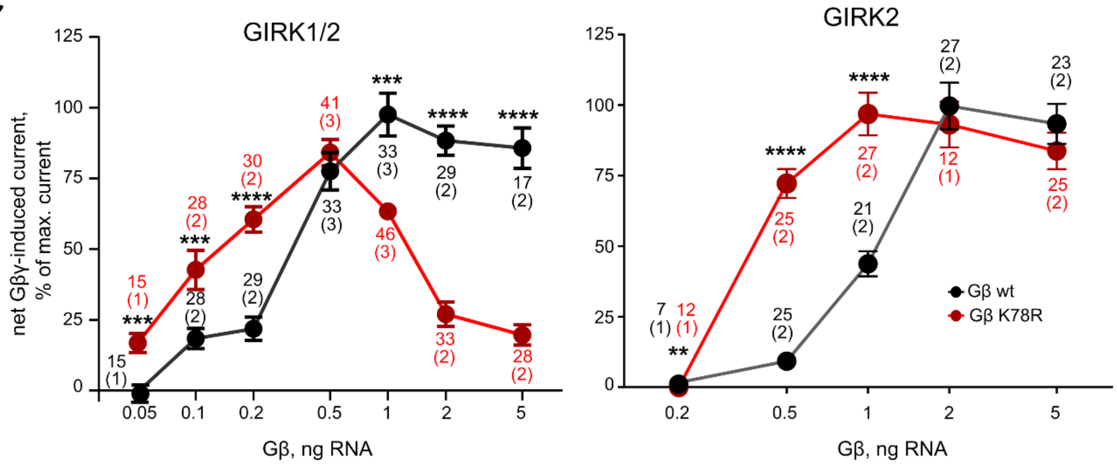

Supplementary Fig. 6. ETX and Gβγ dose-response relationships for GIRK channels expressed in *Xenopus* oocytes. **(A)** Fitting procedures for dose-response dependencies of ETX inhibition of GIRK1/2. Data from **Fig. 7B** were fitted to the indicated equations (solid lines). A standard one-site binding isotherm (gray lines) produced a less satisfactory fit compared to the fit to two-site binding isotherm (red line) or the Hill equation (blue line). **(B)** Parameters of best fits shown in (A). Note that, for GIRK2, the Hill coefficient in the presence of Gβγ and Gβ$_{K78R}$γ is close to one, suggesting the presence of a single population of binding sites with apparent K_d of 25-56 µM. For GIRK1/2, the Hill coefficient (n_H) is substantially lower than 1, suggesting the presence of two binding sites or channel populations with different K_d. Both for GIRK2 and GIRK1/2, the affinity of ETX seems to be

somewhat higher with $G\beta_{K78R}\gamma$ than with $G\beta_{WT}\gamma$. **(C)** Summary of experiments studying the dose dependence of GIRK1/2 (left) and GIRK2 (right) activation by $G\beta_{WT}\gamma$ and $G\beta_{K78R}\gamma$. Net Gβγ-induced currents were calculated in each cell by subtracting I_{basal} of the control group (channel expressed without Gβγ) of the same experiment. To reduce batch-to-batch variability, currents were normalized to the average Gβγ-induced current of the group injected with the highest RNA dose of $G\beta_{WT}\gamma$ in this experiment (2 or 5 ng). Numbers near each experimental point (e.g. 28 (2)) stand for number of oocytes (number of experiments). For each Gβγ RNA dose, currents of the $G\beta_{WT}\gamma$ and $G\beta_{K78R}\gamma$ groups were compared using two-tailed t-test. $^{**}p < 0.01$; $^{***}p < 0.001$; $^{****}p < 0.0001$.

Plate ID		Exp #1	Exp #2	Exp #3	Exp #4	Exp #5	Exp #6	Exp #7	Exp #8	Exp #9
# wells	WT	14	16	11	11	12	8	24	24	24
	K78R/+	17	14	10	11	12	12	24	24	24
nAE	perm_pval	0.051	0.667	0.69	0.751	0.604	0.386	0.387	0.576	0.451
	direction	slight decrease	same	same	same	same	same	same	same	same
MFR/nAE	perm_pval	0.884	0.008	0.096	<0.001	0.467	0.791	0.781	0.01	0.974
	direction	same	decrease	slight decrease	decrease	same	same	same	increase	same
number of bursts/min	perm_pval	<0.001	<0.001	0.001	<0.001	0.001	0.409	0.01	0.051	<0.001
	direction	decrease	decrease	decrease	decrease	decrease	same	decrease	slight decrease	decrease
mean burst duration	perm_pval	<0.001	<0.001	<0.001	0.123	0.004	0.552	<0.001	<0.001	<0.001
	direction	increase	increase	increase	same	increase	same	increase	increase	increase
mean IBI	perm_pval	<0.001	<0.001	<0.001	<0.001	0.003	0.216	0.003	0.036	<0.001
	direction	increase	increase	increase	increase	increase	same	increase	increase	increase
mean spikes in bursts	perm_pval	0.001	0.018	0.005	0.111	0.052	0.334	<0.001	<0.001	0.001
	direction	increase	increase	increase	same	increase	same	increase	increase	increase
mean freq of spikes in bursts	perm_pval	0.237	0.031	0.322	0.025	0.313	0.018	0.008	0.915	<0.001
	direction	same	decrease	same	decrease	same	increase	decrease	same	decrease
% spikes in NS	perm_pval	0.011	0.111	0.179	0.876	0.909	0.022	0.143	0.508	0.869
	direction	decrease	same	same	same	same	increase	same	same	same
Mutual Info	perm_pval	0.444	0.071	0.623	0.036	0.919	0.223	0.585	0.009	0.123
	direction	same	slight decrease	same	decrease	same	same	same	increase	same
STTC	perm_pval	0.001	0.552	0.599	0.978	0.68	0.05	0.665	0.83	0.321
	direction	decrease	same	same	same	same	slight increase	same	same	same

Supplementary Table 1A. Network phenotypes and combined p-values by individual MEA plates. Mann-Whitney U test with 1000 permutations. 9 plates total; 8 biological replicates; experiments #4 and #5 are technical replicates (same cells). The direction indicated is the direction of the change for the Gnb1K78R/+ wells compared to WT ones. nAE, number of active electrodes; IBI, inter-burst interval; NS, network spikes, STTC, spike train tiling coefficient, perm_pval, permutated p-value.

Plate ID	combined p-val MWU after normalization to WT	combined p-val Fisher's test
nAE	0.789<br>same	0.9401776<br>same
MFR/nAE	0.068<br>slight decrease	0.02906075<br>slight decrease
number of bursts/min	<0.001<br>decrease	2.37E-13<br>decrease
mean burst duration	<0.001<br>increase	2.96E-13<br>increase
mean IBI	<0.001<br>increase	9.40E-14<br>increase
mean spikes in bursts	<0.001<br>increase	6.37E-11<br>increase
mean freq of spikes in bursts	<0.001<br>decrease	3.53E-04<br>decrease
% spikes in NS	0.8<br>same	0.2561992<br>same
Mutual Info	0.976<br>same	0.1921119<br>same
STTC	0.681<br>same	0.3102949<br>same

Supplementary Table 1B. Network phenotypes and combined p-values – summary of all MEA plates. Total number of wells: WT 144, K78R/+ 148. The direction indicated is the direction of the change for the Gnb1K78R/+ wells compared to WT ones. nAE, number of active electrodes; IBI, inter-burst interval; NS, network spikes, STTC, spike train tiling coefficient, perm_pval, permutated p-value.

		Inhibitory neurons				Excitatory neurons			
		Mean	SEM	n	p-value	Mean	SEM	n	p-value
V_{rest}, mV	WT	-58.58	0.81	36	0.058	-63.9	1.15	10	0.53
	K78R	-60.61	0.60	31		-63.0	0.78	14	
R_m, MΩ	WT	206.89	17.35	33	0.012	115.85	13.88	13	0.21
	K78R	150.14	15.23	29		113.04	22.48	17	
C_m, pF	WT	73.34	3.91	38	0.03	138.38	13.74	12	0.16
	K78R	87.98	5.36	32		160.25	6.62	15	
AP rheobase, pA	WT	152.35	19.80	34	0.92	123.07	23.31	13	0.096
	K78R	150.90	19.47	33		178.75	25.09	16	
AP threshold, mV	WT	-33.18	1.14	35	0.091	-37.30	1.99	13	0.86
	K78R	-35.97	1.10	30		-37.74	1.48	16	
AP overshoot, mV	WT	21.76	1.55	36	0.148	30.41	2.25	13	0.2
	K78R	25.28	1.81	31		34.34	1.89	16	
AP duration, ms	WT	2.35	0.11	35	0.005	2.05	0.10	13	0.46
	K78R	1.89	0.10	30		1.96	0.05	15	

Supplementary Table 2. Excitability parameters in cultured cortical neurons. AP, action potential
